# Extensive C->U transition biases in the genomes of a wide range of mammalian RNA viruses; potential associations with transcriptional mutations, damage- or host-mediated editing of viral RNA

**DOI:** 10.1371/journal.ppat.1009596

**Published:** 2021-06-01

**Authors:** Peter Simmonds, M. Azim Ansari

**Affiliations:** Nuffield Department of Medicine, Peter Medawar Building for Pathogen Research, University of Oxford, Oxford, United Kingdom; Technische Universitat Munchen, GERMANY

## Abstract

The rapid evolution of RNA viruses has been long considered to result from a combination of high copying error frequencies during RNA replication, short generation times and the consequent extensive fixation of neutral or adaptive changes over short periods. While both the identities and sites of mutations are typically modelled as being random, recent investigations of sequence diversity of SARS coronavirus 2 (SARS-CoV-2) have identified a preponderance of C->U transitions, proposed to be driven by an APOBEC-like RNA editing process. The current study investigated whether this phenomenon could be observed in datasets of other RNA viruses. Using a 5% divergence filter to infer directionality, 18 from 36 datasets of aligned coding region sequences from a diverse range of mammalian RNA viruses (including *Picornaviridae*, *Flaviviridae*, *Matonaviridae*, *Caliciviridae* and *Coronaviridae*) showed a >2-fold base composition normalised excess of C->U transitions compared to U->C (range 2.1x–7.5x), with a consistently observed favoured 5’ U upstream context. The presence of genome scale RNA secondary structure (GORS) was the only other genomic or structural parameter significantly associated with C->U/U->C transition asymmetries by multivariable analysis (ANOVA), potentially reflecting RNA structure dependence of sites targeted for C->U mutations. Using the association index metric, C->U changes were specifically over-represented at phylogenetically uninformative sites, potentially paralleling extensive homoplasy of this transition reported in SARS-CoV-2. Although mechanisms remain to be functionally characterised, excess C->U substitutions accounted for 11–14% of standing sequence variability of structured viruses and may therefore represent a potent driver of their sequence diversification and longer-term evolution.

## Author summary

The rapid evolution of RNA viruses is thought to arise from high mutation frequencies during replication and the rapid accumulation of genetic changes over time in response to its changing environments. This study describes an additional potent factor that contributes to the evolution of RNA viruses infecting mammals, the occurrence of specific C->U mutations in a subset that possess intensely structured genomes. This mutational process substantially damages the virus’s ability to replicate and is potentially akin to “genome editing” of HIV-1 and other retrovirus genome sequences by APOBEC, one of the principal components of vertebrate antiretroviral defence mechanisms. This study however provides evidence for a wider mutational activity against several human and veterinary RNA viruses, including HCV and foot and mouth disease virus. The C->U mutational process accounted for 15–20% of standing sequence variability of these RNA viruses, representing a potent driver of their sequence diversification and longer-term evolution.

## Introduction

The evolution of viruses is typically conceptualised as a combination of adaptive sequence change in response to a range of selection pressures in the environment and a process of random diversification in which neutral or near neutral nucleotide substitutions become fixed in virus populations [1–3]. An extensive literature documents adaptive (or Darwinian) evolution in response to antiviral treatments and escape from host immune responses in the form of T cell and antibody driven epitope escape mutation [4–7]. Viruses may furthermore display a series of changes to encoded viral proteins and even genome rearrangements in the process of jumping hosts and adapting to new internal environments [8,9].

A further source of mutations in viruses arises from effects of several innate antiviral effector mechanisms in vertebrate cells that operate through viral genome editing. Of these, the best characterised are the interferon-inducible isoform of adenosine deaminase acting on RNA type 1 (ADAR1)[10] that targets RNA viruses during replication, and members of the apolipoprotein B mRNA-editing enzyme, catalytic polypeptide-like (APOBEC) family [11]. Although hitherto generally considered as an antiviral pathway primarily active against retroviruses and retroelements, there has been considerable discussion of whether the over-representation of C->U transitions observed in genomic sequences of SARS coronavirus 2 (SARS-CoV-2), rubella virus (RUBV) and potentially other mammalian RNA viruses might originate from a similar process for RNA editing by one or more APOBEC-related deaminases [12–18].

The current study investigated this possibility through construction and analysis of sequences changes in alignments of mammalian RNA virus sequences from a wide range of families. Since APOBEC-mediated RNA editing has been proposed to occur in the context of RNA structure elements, virus datasets were additionally scanned for sequence-dependent internal RNA base-pairing [19,20]. Virus genome structural and compositional factors favouring APOBEC-like C->U editing in these datasets were analysed and the potential contribution of these driven sequence changes to their longer-term evolutionary trajectories estimated.

## Results

### Sequence datasets

The primary resource for the study were aligned sequences from a wide range of mammalian RNA viruses derived from several vertebrate virus families (Tables 1 and S1). These were selected based on the availability of large numbers of complete genome sequences from naturally occurring virus variants collected in previous epidemiological and evolutionary studies. These showed levels of intra-population sequence divergence ranging from 5% - 19% (Table 1). These included viruses with and without large scale RNA secondary structure in the genomes [19–21], with mean folding energy differences (MFEDs) ranging from -0.1% - 17.8% (Table 1). The analysis was supplemented by inclusion of previously described datasets of SARS-CoV-2 and other coronaviruses [21].

**Table 1:** COMPOSITIONAL FEATURES OF RNA VIRUS SEQUENCE DATASETS USED IN THE STUDY^1^.

					Normalised Asymm^4^		
Virus^2^	Family	Polarity	n	MPD^3^	MFED	nG->A	nC->U
BUNV	*Peribunyaviridae*	**-**	92	0.226	0.9%	1.063	0.541
HPeV-3	*Picornaviridae*	**+**	181	0.092	1.6%	1.107	0.938
CHIKV	*Togaviridae*	**+**	245	0.042	5.3%	1.028	0.957
SINV	*Togaviridae*	**+**	101	0.037	1.9%	1.010	1.097
EBOV	*Filoviridae*	**-**	200	0.001	2.2%	1.353	1.118
MeV	*Paramyxoviridae*	**-**	224	0.039	-0.1%	1.189	1.140
HEV	*Hepeviridae*	**+**	100	0.187	3.9%	1.910	1.231
BVDV	*Flaviviridae*	**+**	123	0.178	0.7%	0.950	1.305
IAV_seg1-3	*Orthomyxoviridae*	**-**	1340	0.138	0.6%	0.850	1.309
Porcine_KoV	*Picornaviridae*	**+**	138	0.121	16.9%	0.865	1.387
RABV	*Rhabdoviridae*	**-**	2128	0.129	-0.1%	1.275	1.479
EV-A71	*Picornaviridae*	**+**	1161	0.148	0.7%	0.822	1.509
DENV1	*Flaviviridae*	**+**	1557	0.066	1.8%	0.935	1.575
HPgV-1	*Flaviviridae*	**+**	100	0.122	12.0%	1.181	1.765
RSV-A	*Pneumoviridae*	**-**	100	0.026	1.9%	1.349	1.771
OC43	*Coronaviridae*	**+**	113	0.010	17.7%	1.023	1.851
JEV	*Flaviviridae*	**+**	62	0.088	1.4%	0.924	1.886
MNV	*Caliciviridae*	**+**	63	0.102	7.1%	1.191	2.079
HCV-3a	*Flaviviridae*	**+**	820	0.085	8.7%	0.998	2.110
Canine_KoV	*Picornaviridae*	**+**	25	0.125	17.8%	1.910	2.125
HCV-2a	*Flaviviridae*	**+**	51	0.109	7.7%	1.224	2.149
HKU1	*Coronaviridae*	**+**	27	0.002	9.6%	1.841	2.163
TGEV	*Coronaviridae*	**+**	38	0.022	8.8%	0.782	2.265
FMDV-O	*Picornaviridae*	**+**	246	0.106	11.7%	1.029	2.266
HNoV_GGII	*Caliciviridae*	**+**	100	0.189	1.6%	0.746	2.492
OC43	*Coronaviridae*	**+**	178	0.008	17.5%	1.992	2.503
FMDV-A	*Picornaviridae*	**+**	98	0.112	12.1%	0.997	2.559
NL63	*Coronaviridae*	**+**	61	0.009	8.6%	1.510	2.654
HCV-1b	*Flaviviridae*	**+**	102	0.094	8.5%	1.238	2.905
229E_Camel	*Coronaviridae*	**+**	33	0.002	10.4%	0.720	2.906
229E_Human	*Coronaviridae*	**+**	26	0.007	10.4%	0.842	2.981
MERS-CoV	*Coronaviridae*	**+**	26	0.005	15.7%	1.386	2.986
HCV-1a	*Flaviviridae*	**+**	355	0.083	9.0%	1.441	3.019
RUBV	*Matonaviridae*	**+**	73	5.42%	3.19%	1.258	3.596
SARS-CoV	*Coronaviridae*	**+**	22	0.000	13.5%	0.911	4.177
SARS-CoV-2	*Coronaviridae*	**+**	17550	0.000	15.1%	1.564	7.486

^1^Full metadata is listed in S2 Table

^2^Abbreviations: BUNV: Bunyavirus (*Bunyamwera orthobunyavirus* species); RSV-A: respiratory syncytial virus genotype A; BVDV: bovine viral diarrhoea virus; IAV_seq1-3: mammalian influenza A virus, segments 1–3; HPeV-3: human parechovirus type 3; CHIKV: Chikungunya virus; SINV: Sindbis virus; EBOV: Ebola virus; MeV: measles virus; EV-A71: enterovirus A71; Porcine_KoV: Porcine kobuvirus; RABV: Rabies virus (*Rabies lyssavirus* species); DENV1: Dengue virus type 1; HEV: Hepatitis E virus; HNoV_GGII: human norovirus genogroup II; OC43_gt2: human coronavirus OC43, group 2; JEV: Japanese encephalitis virus; HPgV-1: human pegivirus type 1; MNV: Murine norovirus; HCV-3a: HCV genotype 3a; Canine KoV: Canine kobuvirus; HCV-2a: HCV genotype 2a; HCoV-HKU1: Human coronavirus HKU1; TGEV: transmissible gastroenteritis virus; FMDV-O: foot-and-mouth disease virus type O; HCoV-OC43: human coronavirus OC43; FMDV-A: Foot-and-mouth disease virus type A; HCoV-NL63: human coronavirus NL63; HCV-1b: HCV genotype 1b; 229E_Camel: camel-derived 229E coronavirus; HCoV-229E: human coronavirus 229E; MERS-CoV: Middle East respiratory syndrome coronavirus; HCV-1a: HCV genotype 1a; RUBV: rubella virus; SARS-CoV: SARS coronavirus; SARS-CoV-2: SARS coronavirus type 2.

^3^MPD: mean pairwise uncorrected nucleotide distance

^4^Corrected ratio based on nucleotide composition (see Results text).

### Detection of mutational asymmetries in RNA virus datasets

The previous analysis of the directionality of sequence changes in SARS-CoV-2 and the detection of an excess number of C->U changes was simplified by the minimal sequence diversity of the assembled post-pandemic sequences [22]. SARS-CoV-2 sequence diversity primarily comprised isolated base changes relative to a consensus sequence shared by all but one or a few sequences in the alignment. However, for the datasets analysed in the current study, population diversity was substantially greater making inference of directionality increasingly arbitrary as site variability increased. To overcome this problem, analyses of relative mutation frequencies were restricted to sites showing low degrees of heterogeneity so that the directionality of mutations can be inferred.

**Fig 1:**
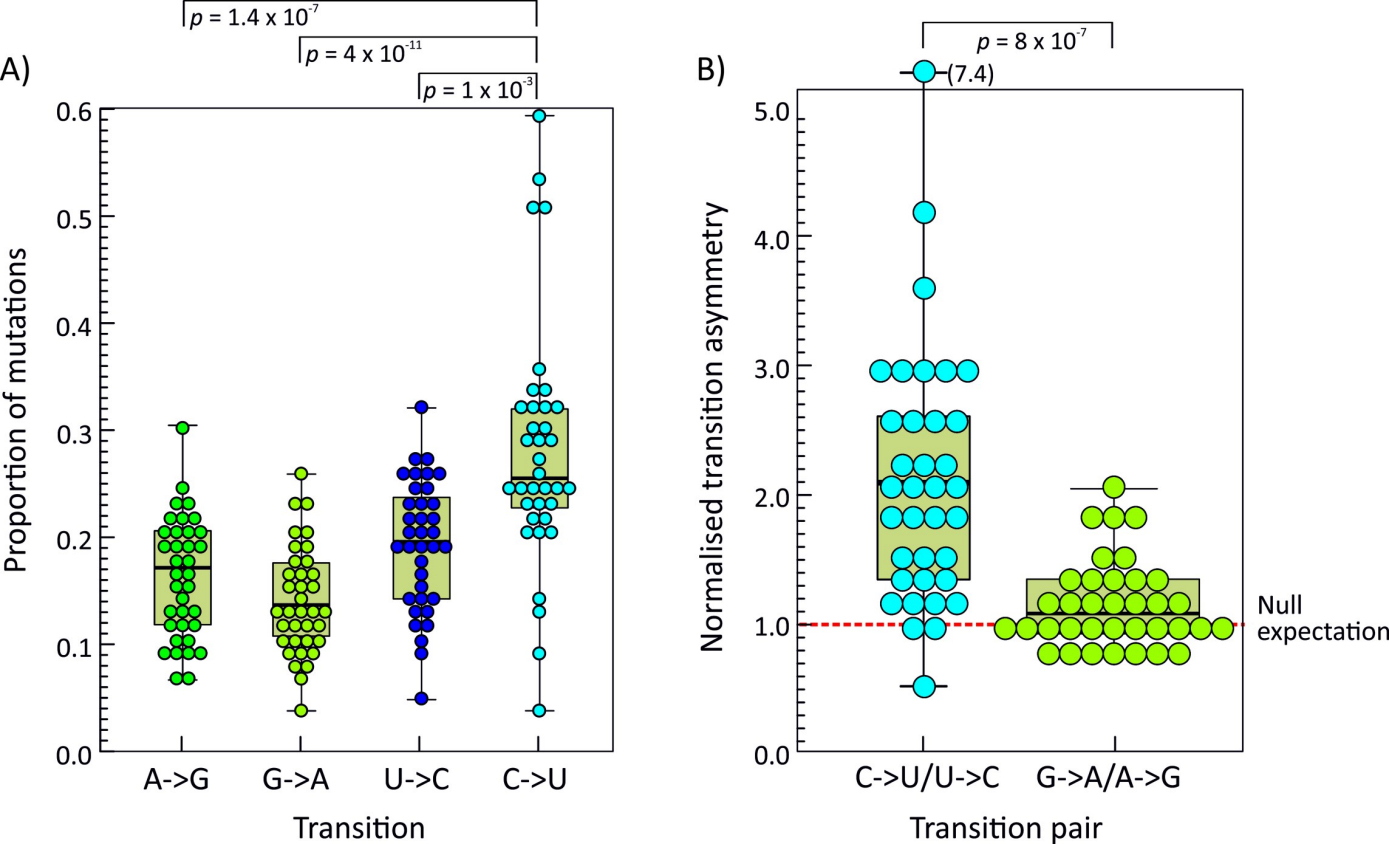
Transition frequencies and asymmetries in RNA virus alignments. Relative frequencies of each mutation type expressed as a percentage of all changes (y-axis) in the 36 RNA virus alignments at sites showing <5% heterogeneity. (B) Comparison of normalised transition asymmetry values; the dotted red line shows the expected unbiased transition asymmetry. For both graphs, distributions were compared using the Mann-Whitney U test; significant (*p* < 0.05) *p* values shown. Box plots show maximum, upper interquartile range (IQR), median, lower IQR and minimum values of each distribution.

Sequence datasets was analysed using the program Sequence Change in the SSE package, which records the occurrences and sites of sequence changes from a majority rule alignment consensus sequence. Collectively, there was a significantly greater number of C->U changes compared to its reverse (U->C) and to other transitions in the RNA virus datasets at sites showing <5% heterogeneity although the degree of over-representation was highly variable between viruses (Fig 1A). Transition frequencies were consistently higher than other mutations (S1 Fig). Transversion frequencies between pairs of bases were comparable, with the exception of a substantially higher frequency of G->U mutations compared to its reverse in coronaviruses but not in other RNA viruses (S1 Fig)

To more formally quantify the degree of over-representation of C->U transitions in each virus dataset, the ratio of each transition to its reverse (Columns 13; 14, S2 Table) was normalised for base composition as described in a previous analysis for SARS-CoV-2 [12] (Columns 7, 8, Table 1). Formally, in the absence of mutational pressure (null expectation), the expected ratio of frequencies of a mutation X->Y to Y->X would be proportional to their native base frequencies and [f(X->Y) / f(Y->X)] / [f(X) / f(Y)] should approximate to 1. Applying this to the observational data in the virus datasets, the high frequencies of C->U changes were reflected in strong C->U / U->C transition asymmetries (Fig 1B; mean value 2.3), again though with a wide range of values from 0.7 (BVDV)– 7.5 (SARS-CoV-2). Contrastingly, the complementary G->A / A->G transition values were less variable and centred around the null expectation (mean 1.2, range 0.6–2.4).

**Fig 2:**
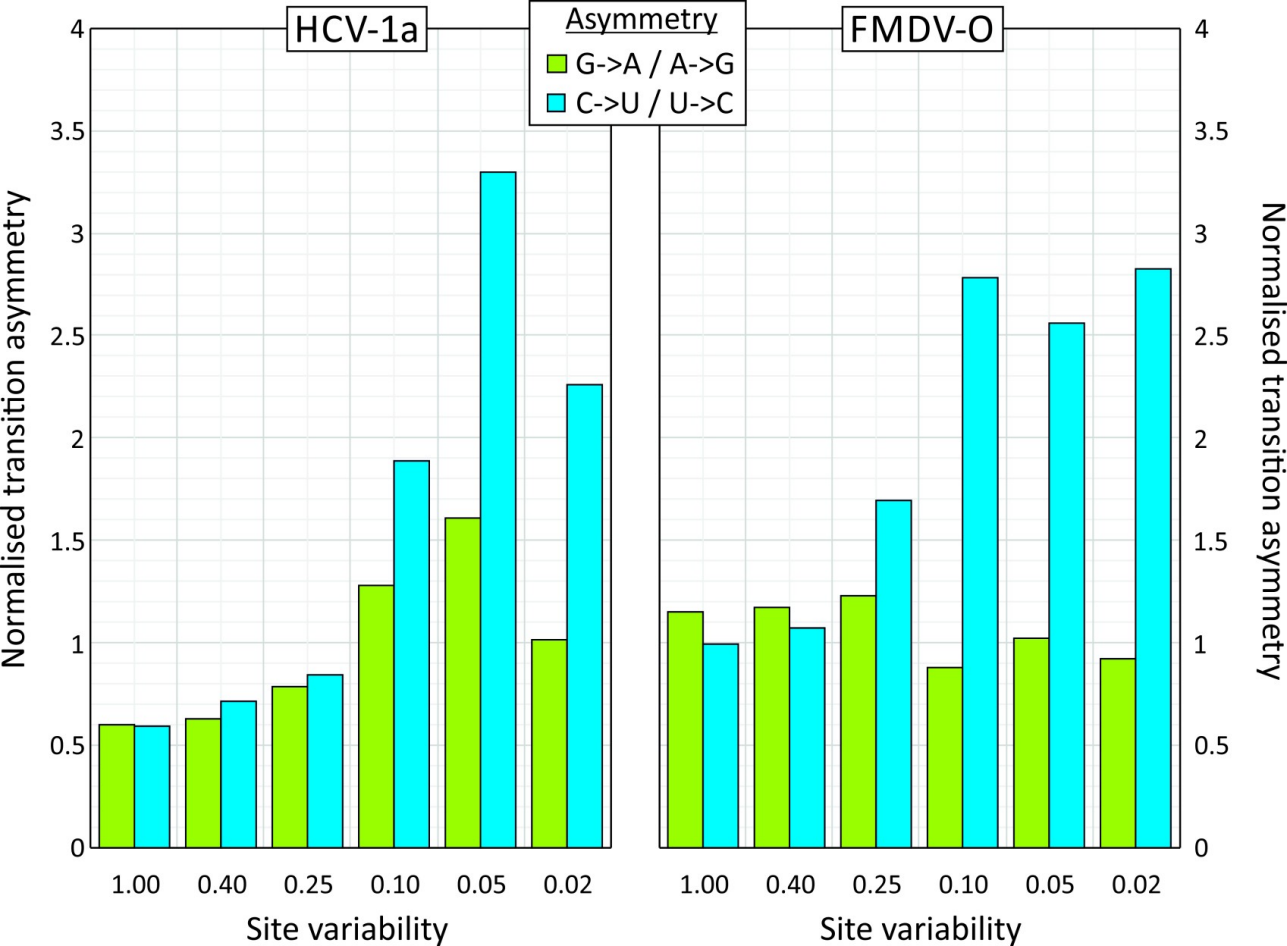
Frequency related transition asymmetries. Transitional asymmetries of two virus datasets showing C->U/ G->A asymmetries. Normalised values (y-axis) were calculated for sites showing different levels of sequence heterogeneity (x-axis): 0.02: 0.02 or less; 0.05: <0.05 and ≥0.02; 0.1: <0.1 and ≥0.05; 0.25: <0.25 and ≥0.1; 0.4: <0.4 and ≥0.25; 1.00: ≥0.4.

This initial analysis of mutational asymmetry was conducted using a 5% heterogeneity threshold to allow directionality of sequences to be inferred. The relationship between site heterogeneity and transition asymmetry was determined for two example RNA virus datasets showing excess C->U changes at the 5% threshold (Fig 2; HCV–mean transition asymmetry 3.0; FMDV: 2.2; Table 1). At highly variable sites, frequencies of G->A / A->G and C->U / U->C were comparable and close to the null expectation. However, for both viruses, increasing asymmetry was observed at sites with reduced heterogeneity, ruling out the possibility that the observed asymmetries were the result of unrecognised compositional biases in the virus datasets, and validating the use of the 5% threshold to analyse directionality of sequence change.

**Fig 3:**
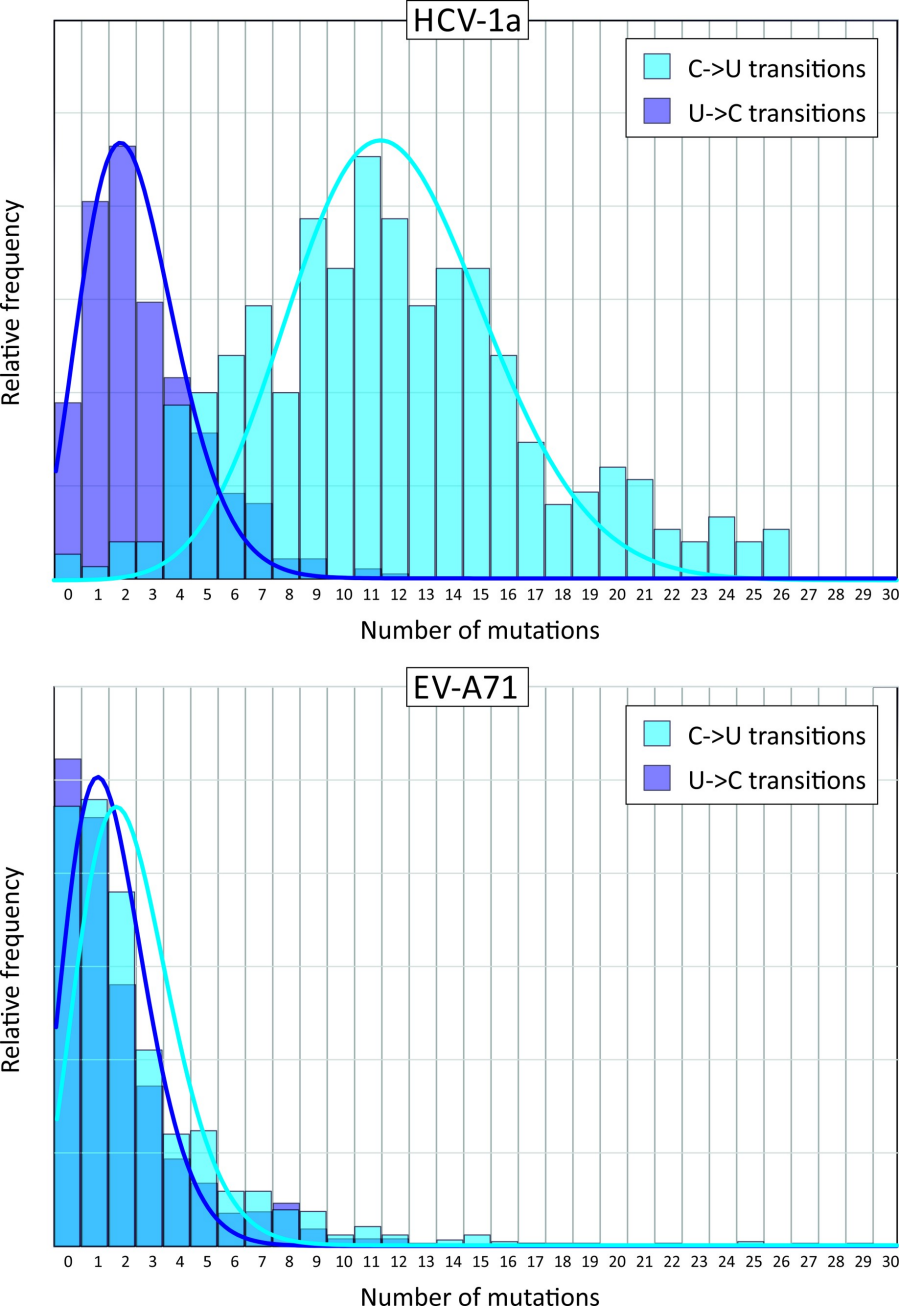
Distribution of C->U and U->C mutations in individual sequences. Numbers of C->U and U->C transitions in individual coding region sequences of HCV-1a and EV-A71 plotted as frequency histograms. Distributions were fitted to Poisson distributions based around their mean numbers of substitutions (light and dark blue lines).

The estimation of relative transition frequencies was collectively based upon all sequences within each virus alignments. To investigate the degree of heterogeneity in C->U changes between sequences, numbers of this transition were computed individually and compared with those of the reverse mutation (Fig 3) in two of the larger datasets showing high and low normalised transition asymmetries (HCV-1a – 3.1 and EV-A71–1.4 respectively; Table 1). For EV-A71, there were means of 3.1 C->U and 2.5 U->C substitutions per sequence at the 5% heterogeneity level, and a distribution of values that approximated to a Poisson distribution, although marginally over-dispersed (Kolmogorov-Smirnov single sample test statistic = 6.8; *p* < 0.001). Similarly for HCV-1a, both U->C and C->U transition frequencies followed marginally skewed Poisson distributions (test statistics 1.5 [*p* = 0.03] and 2.5 [*p* < 0.001] respectively), but with a higher mean number of C->U transitions per sequence (12.1 / sequence) than the reverse (2.8 / sequence). The sequence datasets of HCV and EV-A71 sequences therefore showed no evidence for the occurrence of individual hypermutated sequences as described previously for HIV-1 [23–25]; the driver of elevated C->U frequencies in HCV sequences appeared to operate at a similar intensity on all sequences analysed.

**Fig 4:**
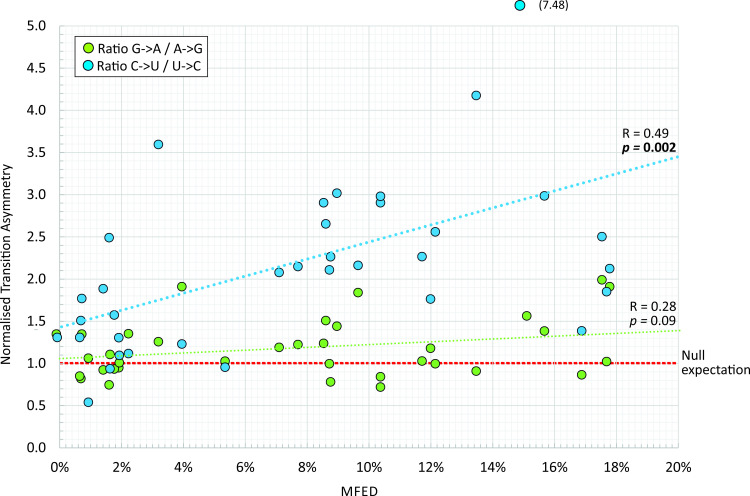
Association of transition asymmetries with RNA secondary structure. The association of transition asymmetry values with MFED values, indicating of the degree of genome RNA folding. Correlation values (R) and significance using linear regression for C->U / U->C and G->A / A->G asymmetries are shown.

To investigate which genome features of RNA genomes were predictive of the C->U/U->C transition asymmetry, a range of compositional attributes (G+C content, representation of CpG and UpA dinucleotides, asymmetry in the number of G bases relative to C, and of A relative to U), mean folding energies (MFEs) of consecutive 300 base fragments and differences of this value from sequence order randomised controls (MFEDs) were computed. The association of each with C->U/U->C and G->A/A->G transition asymmetry values was analysed by multivariate analysis (Table 2). MFED value was the only variable significantly associated with C->U / U->C asymmetry (p = 0.013); this parameter represents the degree of sequence order-dependent RNA folding in the coding region(s) of the virus (Table 2). The association between C->U / U->C asymmetry and MFED was further apparent and readily visualised by simple linear regression (Fig 4; *p* = 0.002). There was no association with MFE, representing the minimum free energy on RNA folding, a property primarily influenced by the G+C content of the sequence, which also showed no association with the C->U / U->C transition asymmetry. There were similarly no associations with extents of CpG or UpA dinucleotide suppression in RNA virus genomes, or base imbalances (U/A, C/G). As expected from the minimal differences from the null expectation, no association between G->A/A->G transition asymmetry values with any compositional or structural sequence attribute was detected.

**Table 2:** PREDICTIVE FACTORS FOR G->A AND C->U TRANSITION ASYMMETRIES^1^ BY ANOVA.

	G->A / A->G		C->U / U->C	
Variable	R^2^	*p*-value^3^	R^2^	*p*-value^3^
G+C	-0.126	0.766	0.089	0.822
C-G_Asymm^4^	0.398	0.089	-0.248	0.322
U-A_Asymm^4^	0.389	0.129	-0.196	0.472
CpG	-0.03	0.924	-0.212	0.512
UpA	0.281	0.208	0.067	0.778
MFE	-0.116	0.766	-0.243	0.55
MFED	-0.092	0.744	0.693	**0.013**
G->A or C->U Asymm.^5^	0.076	0.686	0.082	0.686

^1^Normalised by base frequencies of sites.

^2^Standardized coefficient β.

^3^Significant values below 0.05 shown in bold.

^4^Proportional excess of C bases over G bases, or U over A

^5^Infleunce of alternative transition asymmetry, G->A/A->G or C->U/U->G

### Sequence contexts for C->U transition asymmetry

**Fig 5:**
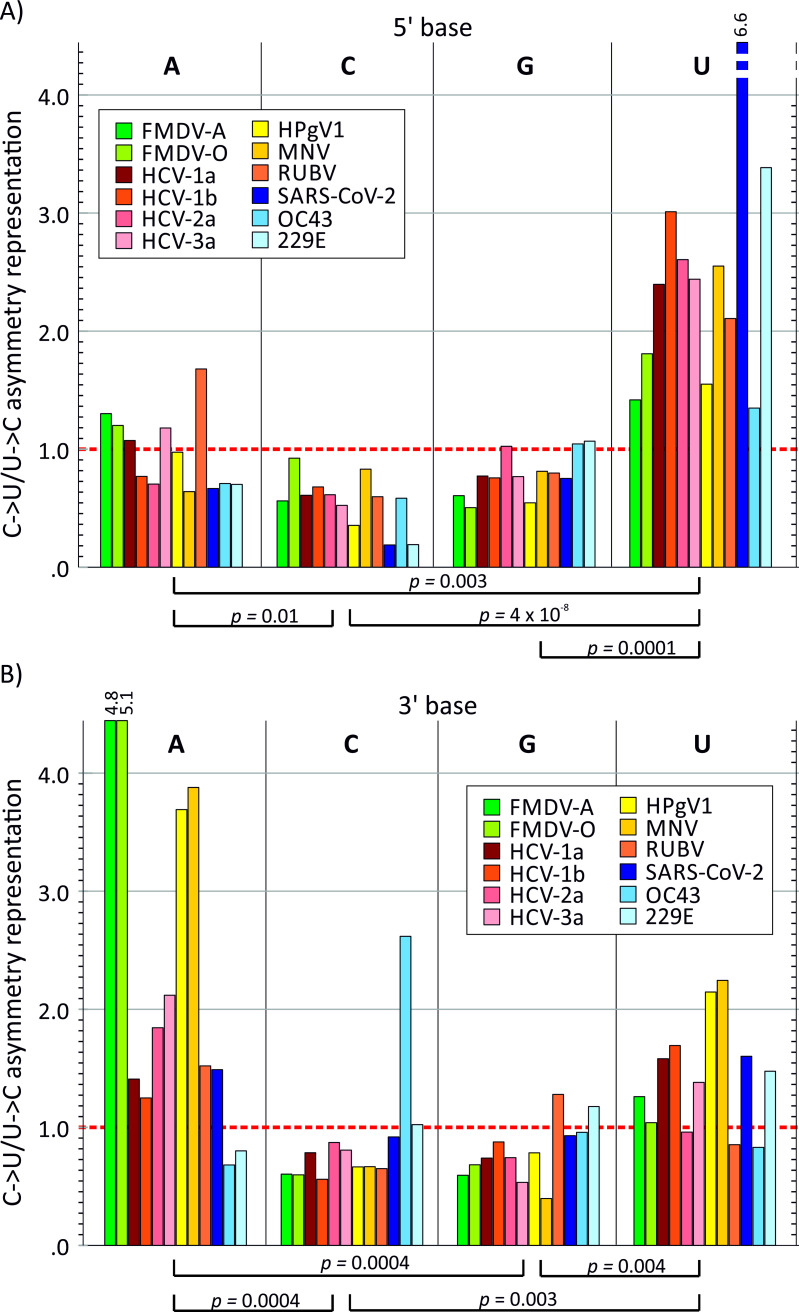
Influence of 5’ and 3’ bases on C->U mutation frequencies. Influence of the identities of the immediate 5’ base and 3’ bases on C->U mutation frequencies in a range of RNA viruses showing C->U/U->C transition asymmetry. Normalised C->U/C->U transition asymmetries in each 5’ and 3’ context were adjusted to account for 5’ or 3’ base frequencies. The y-axis shows the over- or under-representation of the asymmetry values in each context relative to the value for all contexts; the null expectation (no effect of 5’ or 3’ base) was 1.0 (red dotted line). Distributions of values for each context were compared by Mann-Whitney U test; *p v*alues < 0.05 shown.

C->U transitions in SARS-CoV-2 genomes were influenced by the immediate 5’ and 3’ base contexts of the mutated site [12]. Other RNA virus datasets showing C->U / U->C transition asymmetries were analysed similarly (Fig 5). Once normalised to base composition (S3 Table), relative mutation frequencies varied over a substantial range but with a 5’U being consistently associated with greater C->U/U-C transitional asymmetry. Sites with a 5’U showed a mean over-representation for all viruses of 2.0 compared to 0.78, 0.56 and 0.84 in 5’ A, C and G contexts respectively (*p* values of 0.0004, 5 x 10^−11^ and 8 x 10^−5^ respectively by Mann Whitney U test). Effects of 3’ context were far more variable between viruses, but with evidence for favoured C->U transitions upstream of A in FMDV and to lesser extents in other RNA viruses.

### Homoplasy of C->U mutations

Previous analyses demonstrated that a proportion of C->U mutations in SARS-CoV-2 failed to become genetically fixed in a population [26]. The distribution of many mutations violated the overall phylogeny of the dataset, appearing convergently and transiently in different parts of the tree [12]. The possibility that excess C->U changes observed in current datasets might be similarly homoplastic was investigated more systematically through measurement of the concordance between nucleotide identities at variable sites in virus alignments and their overall tree topology. This enables segregating substitutions that reflect evolutionary relationships to be distinguished from phylogenetically uninformative or incongruent sites that may arise from host-driven mutational processes. As the method does not require directionality to be inferred, the approach is not restricted to relatively invariant sites (<5% heterogeneity) examined in previous analyses.

**Fig 6:**
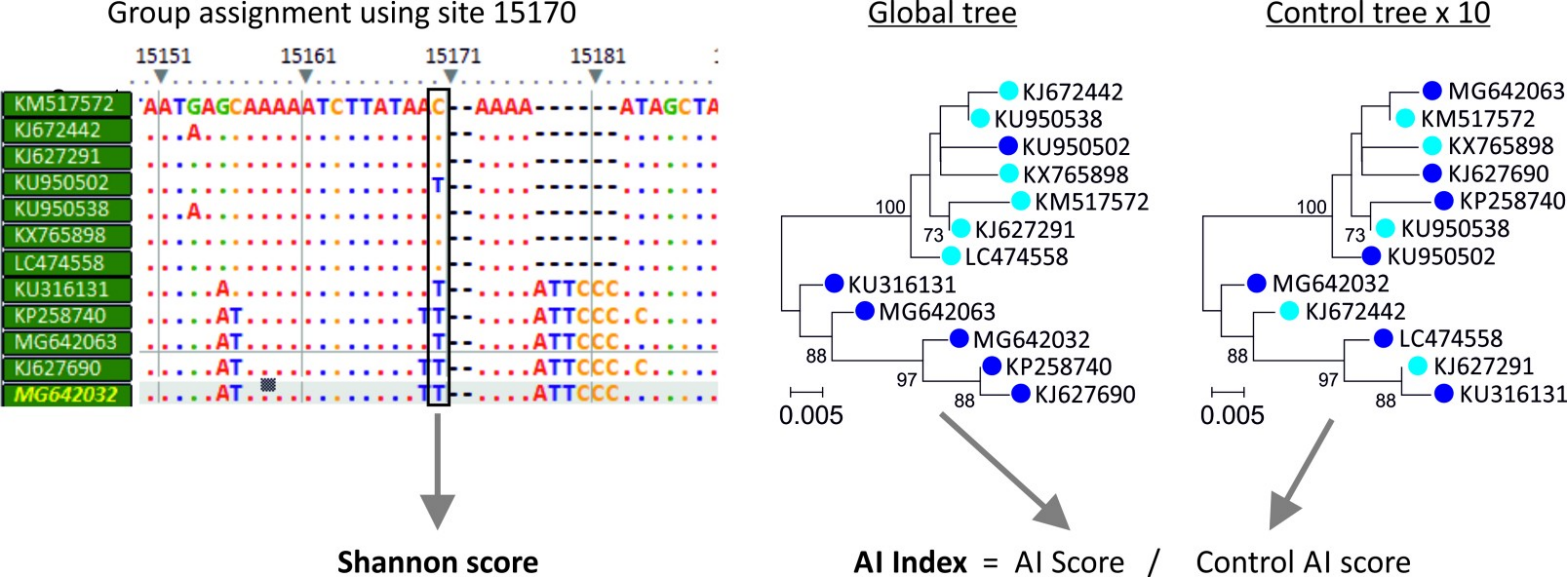
Using the association index to determine informativeness of individual sites. Schematic summary of the steps used to investigate site informativeness. Individual alignment positions are sequentially analysed for their concordance to a global phylogeny. Base identity is used to assign groups which are then use for calculation of an association value through group segregation in a neighbour-joining tree of the alignment where non-bootstrap supported branches are collapsed. The AI index is its ratio to the mean association value of 10 sequence label order randomised controls (representing the null expectation of no association). Finally, the Shannon entropy score, representing site heterogeneity is recorded.

**Fig 7:**
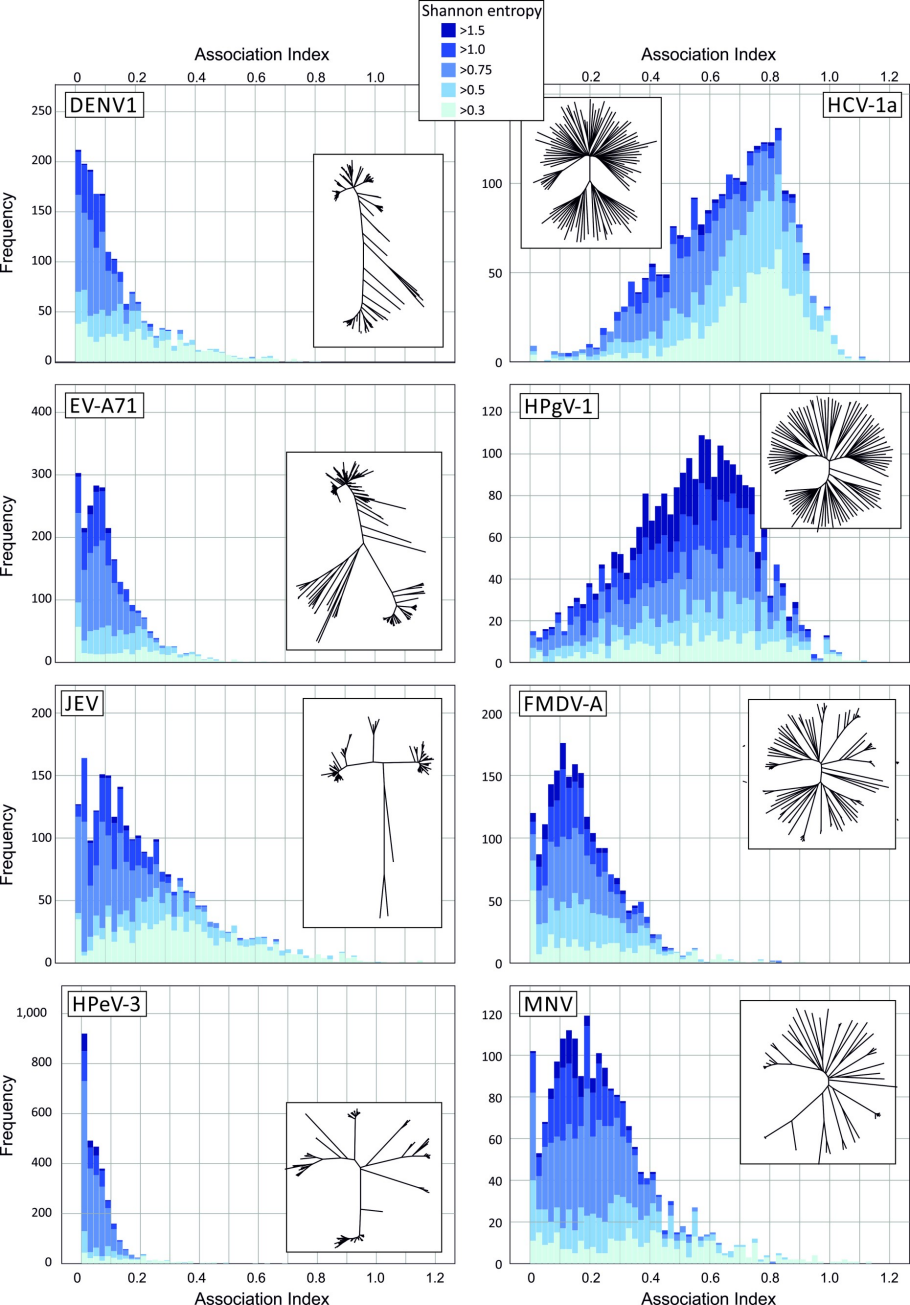
Distribution of association index values in virus datasets. Frequency distributions of AI values at variable sites in alignments of representative viruses showing unbiased (left) or elevated (right) C->U/U->C transition asymmetries and with comparable overall sequence divergence (MPD values listed in Table 1). Histograms were sub-divided based on their Shannon entropy range (see key for colour coding; minimally variable sites (Shannon entropy < 0.3) were excluded). Insets show the corresponding tree topologies for each virus analysed, for large datasets (HCV, EV-A71; DENV1), trees based on randomly selected representative sequences are shown for clarity. Phylogenetic trees drawn to scale are provided in S2 Fig.

The program, Homoplasy Scan in the SSE package was developed to sequentially analyse each variable site in a virus sequence alignment; this recorded the degree of segregation of each base in a global tree constructed from 1200 base genome fragment that incorporates the interrogated bases (Fig 6). Association index (AI) values based on sequences grouped by their component bases were typically low in DENV3, HPeV-3 and EV-A71, indicating that most substitutions co-segregated with overall phylogeny. AI distributions were typically narrower (more informative) for more variable sites (high Shannon entropy values) despite the potential effect of site saturation and convergence on site with only 4 possible character states. The pattern of base segregation was remarkably different in alignments of HCV (results from genotype 1a are shown but other genotypes were closely similar and HPgV-1 (Fig 7). In these, only a small fraction of sites showed a base distribution that segregated with (and defined) the overall phylogeny of the alignment; the majority, irrespective of their underlying diversity, poorly matched overall phylogeny with AI values approaching the mean of the null distribution (AI = 1.0). Distributions for FMDV, MNV and JEV were intermediate between these extremes.

Broadly, the observed differences arose from different genetic structuring of sample populations; phylogenetic trees from alignments with predominantly informative sites showed a marked degree of structured internal branching (S2 Fig), while variants of HCV and HPgV-1 showed deep branching and little ordering of sequences beyond their initial diversification. The differences in tree structure between datasets were quantified using a lineage through time plot generated by the LTT program in the Phylocom package [27] (S3 Fig). This depicts the substantial lineage diversification of HCV, HPgV-1 and to lesser extents of MNV and FMDV-A at the base of the tree, and a contrasting late diversification of JEV, DENV1, HPeV-3 and EV-A71.

**Fig 8:**
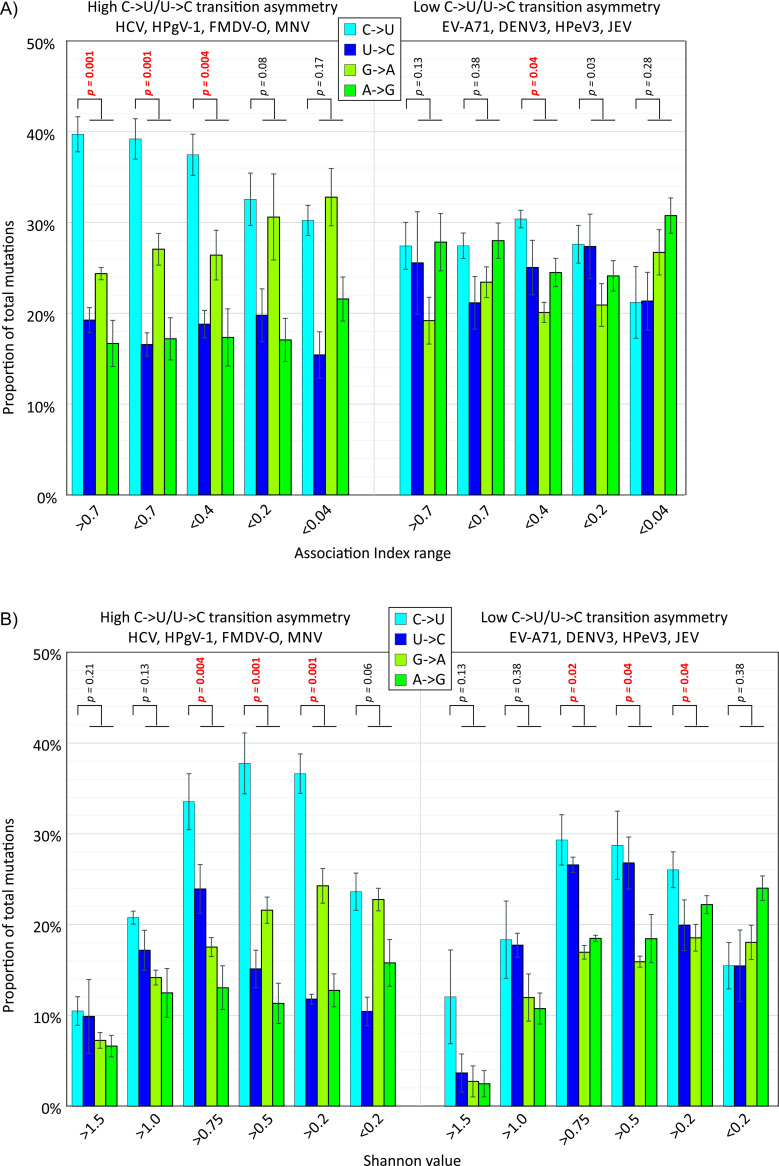
Effect of association index values and site variability transition frequencies. Relative frequencies of different transitions at sites varying in AI value, reflecting their phylogenetic informativeness (A), and in sequence heterogeneity (B). Bar heights show means of the four component virus datasets; error bars show standard errors of the mean). Frequencies of C->U were compared with frequencies of the other three transitions in each band using the Mann-Whitney U test; significant values shown in red.

Irrespective of the actual distributions of AI values (and associated differences in tree topologies of the different RNA virus datasets), categorisation of sites based on AI value ranges allowed an investigation of whether C->U transition frequencies were specifically over-represented at phylogenetically uninformative sites as would be expected if these mutation were subject to homoplasy (Fig 8). Amongst representative viruses showing C->U/U->C transition asymmetry (HCV-1a, HPgV-1, MNV and FMDV-O), there was a significant excess of C->U transitions compared to other transitions at uninformative sites (AI values > 0.2; Fig 8; upper left graph), in contrast to ratios observed in the unbiased dataset (EV-A71, JEV, DENV1 and HPeV-3; upper right graph). Excess C->U transitions were also more extensively distributed at sites of medium / low Shannon entropy values (lower left graph). Contrastingly, the representation of other transitions (U->C, G->A and A->G) showed no association with either AI score or site variability. The relationship between excess C->U changes at sites with high AI values and low variability is consistent with the hypothesis for extensive homoplasy specifically of this transition.

### Contribution of C->U transitions to viral diversity

**Fig 9:**
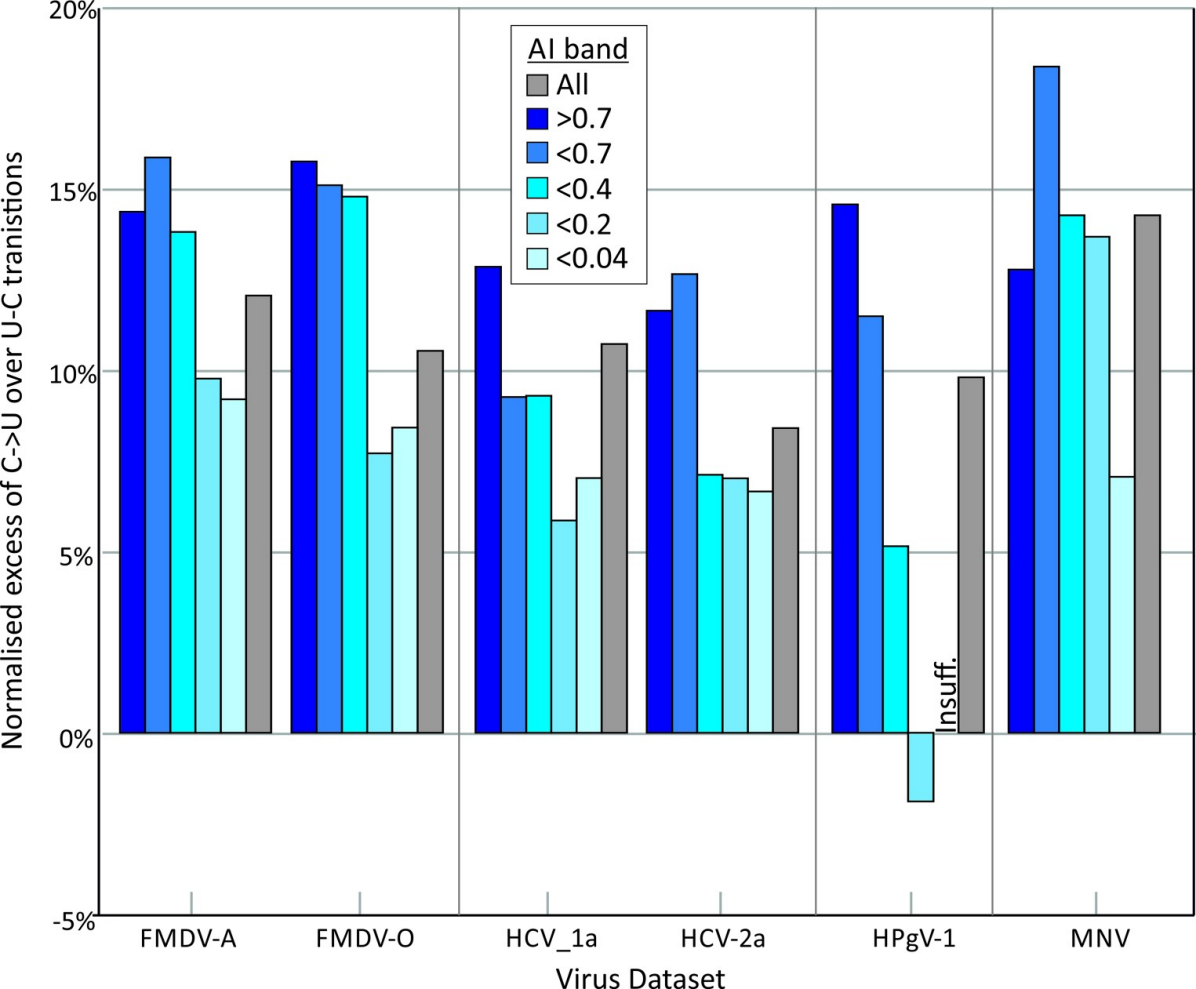
Proportionate excess of C->U over U->C transitions in phylogenetically informative and non-informative sites. Excess C->U mutations (number of sites with majority C->U transition–sites with U->C) expressed as a proportion of all variable sites in genome alignments of viruses showing C->/U->C asymmetry. Proportions were normalised by mononucleotide base frequencies. Separate proportions were calculated by AI band, representing sites that were phylogenetic informative (low AI values) through to uninformative (high AI values).

The extent to which the observed excess of potentially homoplastic and transient C->U mutations in certain viral datasets contributed to overall viral sequence diversity was calculated. The number of sites in a virus alignment showing a majority C->U change subtracted by those showing majority U->C changes (excess C->U) were expressed as proportion of the number of variable sites in the alignment, with totals sub-divided into sites showing different ranges of AI values (Fig 9). There was a substantial over-representation of sites showing excess C->U mutations in HCV, HPgV and other virus datasets showing the C->U / U->C asymmetry, particularly at sites with high AI values. From these, it appears that the asymmetric mutational process contributes a substantial proportion of their standing viral diversity in all four of the viruses analysed (11% - 14% of variable sites).

## Discussion

### Mutational asymmetries in RNA viruses

This study provides evidence for an excess of C->U changes over the reverse (U->C) and complementary (G->A) transitions in a diverse range of RNA viruses, including HCV (all genotypes), FMDV, MNV, HPgV-1 and rubella virus. Fold excesses ranging from 1.9x – 3.6x (Table 1 and Fig 3A) approached those reported in previous analyses of SARS-CoV-2 (7.5x), SARS-CoV (4.2x), MERS-CoV (3.0x) and were comparable to those reported in seasonal coronaviruses (1.8x – 3.0x) [12]. There has been little systematic investigation of the phenomenon of this type of mutational asymmetry in RNA viruses, although it has been long recognised that an RNA editing enzyme, ADAR1 may play a role in prominent excess of U->C and A->C mutations in measles virus genomes associated with sub-acute sclerosing panencephalitis (SSPE) [28], while APOBECs have been shown to create hypermutated proviral DNA copies of HIV-1 and other retroviral genomes [23,29,30]. A recent study identified a noticeable excess of C->U changes in the genomes of rubella virus persisting in patients after immunisation [14,31] a finding that was replicated using the analytical methods of the current study on that dataset, where analysis of a larger dataset of circulating rubella virus strains (n = 73) recorded an even higher C->U / U->C transition asymmetry (3.6x; Fig 4). Although the authors linked the asymmetry to RUBV persistence [14,31], perhaps by analogy with measles virus and SSPE, the finding of substantial C->U asymmetry in circulating RUBV strains in the current study indicates this phenomenon occurs as part of a natural transmission chain of rubella virus infections.

### Mutational mechanisms

The underlying mechanism(s) for the observed elevated frequencies of C->U changes in certain RNA viruses are functionally uncharacterised and conceivably may originate through several mechanisms. In the discussion, we will briefly review the evidence for or against transcriptional, RNA damage-associated and cellular RNA editing mechanisms that may create the excess C->U mutations observed in RNA viruses, taking into account their prominent (+)strand asymmetry, 5’ base context effect and their variable distributions in viruses with structured and unstructured genomes.

#### RNA transcription

A major source of mutations in viruses and other organisms are transcription errors by cellular RNA or DNA polymerases that are incorporated during replication. Different types of polymerases may show varied mutational profiles with separate propensities to mis-incorporate particular transitions or transversions. Very limited data exists on mutation frequencies associated with viral RNA dependent RNA polymerases (RdRps). However, biochemical assays of misincorporation kinetics on RNA templates by the poliovirus RdRp [32,33] or retroviral reverse transcriptase [34] did not identify C->U misincorporation to be favoured over other mutations.

Mutations introduced by RNA transcription would be expected to be symmetric in RNA viruses as their genomes derive from an equal number of positive and negative strand copyings by the same RdRp operating in the same cellular compartment. A tendency to misincorporate a U instead of a C would therefore be reflected in a parallel number of G->A mutations where it occurred on the minus strand. However, as observed previously for SARS-CoV-2 [12–14,26], the frequency of G->A mutations was substantially lower than C->U changes, and generally comparable to those of the other transitions, A->G and U->C (Figs 1 and 2).

To counter this, it could be argued that natural selection operates differently on the minus- and plus-RNA strands where the limited number of minus-strand templates produces a much larger number of genomic RNAs. Therefore there may be more stringent selection against mutations occurring in the negative-strand as these are invariably copied into the plus-strand, whereas plus-strands are relatively dispensable and mutations occurring during their synthesis will not overly affect the overall replication process. Indeed, full-length transcripts with plus-strand synthesis errors could be readily packaged into virions assembled from viral proteins synthesised from independently transcribed mRNAs.

An alternative possibility is that mutations occurring during transcription of the minus strand are more rather than less likely to be fixed because minus strands are in a substantial minority of viral RNA sequences in the infected cell. Mutations arising from specific RdRp misincorporation biases occurring early in infection during synthesis of the minus-strand might be more likely to become fixed in the infected cell through a founder effect than mutations occurring from the 10s or 100s of plus-strands synthesised from that template. In contrast to the selection-based asymmetry hypothesis above, the observed excess of C->U mutations in the genomic (plus-)strand must therefore have originated from elevated frequencies of G->A mutations in the minus-strand rather than C->U.

Neither hypothesis for a transcriptional origin of the C->U strand asymmetry recorded in the current study is supported by the observation of its variable presence in different RNA virus groups and association with genomic RNA secondary structure. While it is possible that RdRps from different RNA viruses may vary in their spectra of misincorporation frequencies, it is notable that C->U asymmetries cut across family divisions, with examples of picornaviruses and flaviviruses showing examples of viruses with both biased (HCV, FMDV, kobuviruses) and unbiased (BVDV, EV-A71, HPeV-3) C->U transition frequencies. The association of C->U asymmetry with the presence of genomic RNA secondary structure (Fig 4) therefore does not mechanistically support a role of differentially selected RdRp mutational errors or founder effects as the explanation for the observations. The RNA secondary structure association is particularly problematic for a transcriptional origin of the C->U asymmetry since its effects are only manifest on the single-stranded genomic viral RNAs, and not within the primarily double-stranded RNA replication complex where the proposed RNA transcriptional mutational errors occur. Secondly, the observed preference for a 5’U at C->U mutated sites (Fig 5) is not supported by what is known about potential conditioning effects of transcriptional contexts influencing RdRp error rates [32,33].

#### Reactive oxidative species (ROS)

It has been recently proposed that substitutions occurring in the SARS-CoV-2 genome, notably the G->U transversion may originate from mutational damage to viral RNA during periods of oxidative stress [15,16]. ROSs are widely associated with DNA mutations, particularly those that target guanine [35]. The oxoguanine bases formed are copied as adenine, creating G->T transversions. As ROSs may also target single-stranded RNA, the production of ROSs on viral infections [36] may potentially account for its previously described over-representation of G->U tranversions in SARS-CoV-2 sequences [15–17]. We found that higher frequencies of G->U compared to reverse (U->G) or complement (C->A) mutations were more widely found in coronaviruses but broadly absent in the other RNA viruses analysed in the study (S1 Fig). The presence of ROS is not clearly associated with damage to other bases, such as modifications of cytidines that would template the observed excess of C->U transitions.

#### RNA editing

An alternative source of mutations arises from the documented effects of several innate antiviral effector mechanisms in vertebrate cells that operate through a process of genome editing; these may potentially introduce mutations into RNA virus genomes during replication. Of these, the best characterised are the interferon-inducible isoform of adenosine deaminase acting on RNA type 1 (ADAR1)[10] that targets RNA viruses during replication, and members of the apolipoprotein B mRNA-editing enzyme, catalytic polypeptide-like (APOBEC) family [11]. The substrate for ADAR1 is double-stranded (ds) RNA formed as a replicative intermediate; upon binding, it catalyses the deamination of adenine bases to inosine which are subsequently copied as a G by viral RNA polymerases, creating A->G base mutations (or U->C on the opposite strand). Although ADAR1 activity has been widely proposed as a mutational mechanisms in SARS-CoV-2 [13,15,16] and other RNA viruses (reviewed in [37]), the direction of changes induced (U->C, A->G) is opposite to what is predominantly observed in virus datasets analysed in the current study.

Members of the APOBEC family typically target single stranded DNA templates for mutagenesis of cytidine to thymidine during reverse transcription of retroviruses and hepatitis B virus (HBV) and in the genomes of small DNA viruses such as papillomaviruses [11,38,39]. For example, APOBEC3G editing of the single stranded DNA generated by reverse transcription of retroviruses generates a damaged proviral copy unable to direct further retroviral replication [29,30,40]. There has been extensive discussion over whether the observed over-representation of C->U transitions in SARS-CoV-2 is driven by one or more of the APOBEC proteins [12–18]. The current study shows a similar over-representation of C->U changes in the subset of RNA viruses that possessed structured genomes, including other coronaviruses; these findings would therefore predict a strikingly wider breadth of the antiviral activity of this pathway than is currently recognised.

If APOBEC was responsible for the over-representation of C->U changes in RNA virus genomes, there should be an increased C->U/U->C mutational asymmetry downstream of a U, as this is the target preference of most mammalian APOBEC paralogues. Consistently with findings of cellular mRNA editing by A3A [41,42] and proposed for rubella virus [31], C->U transitions with a 5’U context showed a 1.5–3 fold increased representation compared to other upstream bases (Fig 5A), with particularly high values in SARS-CoV-2 as previously described [12–14]. However, there was additionally an apparent favoured downstream context (3’A > 3’U > 3’C/G) among the majority of RNA viruses analysed (Fig 5B), similar to what has been described for SARS-CoV-2 mutational preferences but not generally recorded for retrovirus or other APOBEC target sequences [43].

The wide range of G+C contents of the viruses analysed in the current study (Table 1) necessitated the calculation of normalised context representations to enable meaningful comparison of 5’ and 3’ base preferences between different RNA viruses. While there any many ways to achieve this, we adopted the simple approach of first calculating normalised C->U/U->C ratio in each 5’ or 3’ context and then calculating their relative representations taking into account their global mononucleotide frequencies (see Materials and Methods). This yielded relatively consistent over-representations of C->U in a 5’U context that was less apparent in non-normalised data (S3 Table). Other approaches might include normalisation based on dinucleotide frequencies (*eg*. the relative representation of UpC, ApC, GpC and CpC) to normalise for 5’ compositional effects. However, the problem with this approach is determining 3’ contexts since the CpG dinucleotide is substantially suppressed in mammalian +strand viruses, often to 15–20% of expected frequencies [44–46]; attempts to normalise this typically lead to a substantial compensatory and potentially artefactual over-representation of this context that are unlikely to represent the true editing preferences of APOBEC.

The favoured 5’ context of APOBEC-mediated mutations and consequent depletion of TpC/UpC dinucleotides has been exploited as a means to identify editing “footprints” in viral genomes in a previous bioinformatic analysis [47]. Depletion of TpC was detected in a wide range of small DNA viruses, particularly polyomaviruses, parvovirus B19V, herpesviruses. Amongst RNA viruses, UpC depletion was only detected in some seasonal coronaviruses that show substantial genome wide enrichment of U and depletion of C previously ascribed to cytidine deamination [48,49]. The relative weakness of the 5’U context effect on C->U transition frequencies in other RNA viruses (1.5-3-fold for most RNA virus datasets; Fig 5) may explain why TpC depletion was not readily detected by this method.

Apart from 5’ and 3’ base contexts, the only other compositional metric influencing the extent of C->U / U->C transition asymmetry was RNA structure formation (Table 2), a genomic property of a subset of +strand RNA viruses displaying the previously described genome-scale ordered RNA structure [19,20,50]. There was a significant association between MFED value and C->U / U->C asymmetry ratio (Table 2 and Fig 4), but no association with the G->A / A->G normalised asymmetry values.

This association with structured RNA virus genomes potentially recapitulates the previous noted restriction of editing of human mRNA sequences by A3A to sites in defined stem-loop contexts [41]. In an analysis of human transcriptome RNA sequences, over half of the identified edited sites were flanked by short palindromic sequences, typically locating the edited base in the terminal unpaired region of a stem-loop. Supporting this structural association with APOBEC editing, an analysis of the contexts of SARS-CoV-2 and rubella C->U edited sites identified preferential C->U mutations in terminal loop compared to stem sequences [14], or in predicted unpaired compared to unpaired regions [21]. The observed association of excess C->U changes in HCV and other structured viruses is therefore consistent with the action of one or more APOBEC isoforms directly editing a wider range of RNA virus genomes.

While these associations are intriguing, the evidence is circumstantial without systematic functional studies to support a role of APOBEC-mediated RNA editing in restriction of RNA virus replication. Indeed, it was shown that antiviral effects of APOBEC occurred in the absence of any evidence for RNA editing of coronavirus HCoV-NL63 genomic sequences [51]. It would be also unclear where and how editing of RNA virus genomes might occur in the infected cell. The strand-specificity and its presumed occurrence on +strand RNA sequences suggests that editing takes place on naked genomic RNA, perhaps during virus entry or during packaging, rather than co-transcriptionally as documented for retroviruses. Whether and in what cytoplasmic location genomic RNA may be exposed to APOBEC is unclear; coronaviruses show marked excesses of C->U changes, but their genomes, like those of–strand RNA viruses, are typically associated with ribonucleoprotein throughout the replication cycle and must substantially limit their exposure to host pattern recognition receptors.

Inferences on mechanism based on bioinformatics analyses therefore must at this stage be indirect; they should additionally acknowledge that the currently recognised repertoire of RNA editing pathways and other mutational mechanisms in mammalian and other vertebrate cells is almost certainly incomplete. Viral RNA editing may indeed originate from an entirely different mechanism outside the current ADAR1 and APOBEC paradigm. As recently suggested, overlapping but nevertheless distinct C->U mutational contexts in SARS-CoV-2 and rubella genomes points towards the operation of more than one mutational mechanism [14]; the heterogeneity in the effects of 3’ base contexts (Fig 5) may be further evidence for the existence of more than one pathway. Establishment of effective methods to induce and quantify RNA virus editing *in vitro* and a targeted gene deletion approach to functionally test editing abilities of individual APOBEC proteins on RNA templates would be important steps in such investigations.

### Evolutionary consequences of C->U hypermutation

Irrespective of the underlying mutational mechanisms, the analyses performed in the study provided evidence that a substantial proportion of population variability in HCV and other structured viruses could be attributed to a marked over-representation of C->U transitions (15% - 20% of total sequence changes; Fig 9). The observed substitutions correlated poorly with overall phylogeny of viruses in the alignment based on association index calculations (Fig 7A), consistent with previous analyses that documented extensive homoplasy of C->U changes in SARS-CoV-2 [12,26]. The limitations of this type of analysis should however be acknowledged. While the association index calculation represent an established and robust, phylogeny-based method for evaluating group membership with phylogeny [52,53] and performs well compared to other metrics of genetic partitioning [54], analyses in the current study were based upon groupings derived from base identities. These are necessarily limited to between two and four character states defining groups at each alignment position, and this restriction may create mutational saturation effects at highly variable sites and lead to false detection of homoplasy. However, even sites with relatively low Shannon entropy values (0.3–0.5), that would not create any consistent saturation effect showed an excess of C->U changes in HCV and HPgV-1 alignments (Fig 8).

The distribution of C->U changes (and other transitions) at an individual sequence level approximated to a Poisson distribution (Fig 3), albeit with some over-dispersion in the EV-A71 sequence dataset likely arising from its phylogenetic structuring (S3 Fig) compared to HCV. There was no evidence for the occurrence of individual hypermutated sequences in a background population of non-mutated sequences, as previously observed in HIV-1 and hepatitis B virus (HBV) [23–25,55,56]. This likely originates from the differences in replication cycles of RNA viruses and retro-transposing viruses–the RNA virus sequences in the current study were derived from consensus sequences and therefore would have to be fully replication competent and evolutionarily fit to be represented in clinical samples. In contrast, hypermutated sequences of HIV-1 are typically derived from integrated proviruses derived from APOBEC-edited reverse transcription. Their survival in memory T cells occurs irrespective of whether they are able to generate infectious virus or not; similarly for HBV [55,56]. Such sequences can therefore accumulate extensive and bizarre mutational damage as they are effectively evolutionary dead-ends. In marked contrast, the excess of C->U changes observed in structured RNA virus genomes may therefore represent the maximum tolerable mutational load compatible with viability and onward transmission.

While tangential to the primary focus of the study, the shape of phylogenetic trees constructed from different RNA viruses differed substantially (Figs 8 and S2) and potentially contributed to observations of homoplasy. These differences in branching density have been previously quantified using the temporal clustering (TC) metric [57]. The bush-like, over-dispersed topology of HCV showed a lower TC value that derived from a neutral evolutionary simulation, a difference attributed to potential rate variation in different lineages of HCV or population subdivision which promotes the co-existence of lineages. The latter model may potentially be equated with distinct patterns of endemic and epidemic partitioning in the different trees associated respectively with persistent and non-persistent virus infections (S2 Fig). However, there is the further possibility that tree shape and the associated occurrence of phylogenetically uninformative sites in structured virus genomes may also be influenced by extensive RNA editing and homoplastic cycles of mutation and reversion as observed in SARS-CoV-2 [12,26]. The development of evolutionary simulation methods where RNA editing is incorporated and parameterised may lead to valuable insights into the nature and trajectory of short-term diversification. It may serve to better characterise the evident differences between RNA viruses in the nature of their divergent evolution. The observation that excess C->U changes accounted for 11%-14% of variable sites of HCV, HPgV-1, FMDV and MNV at any one time (Fig 9) indicates the powerful role of C->U hypermutation in the generation of RNA virus diversity.

## Materials and methods

### Sequence datasets

Alignments of sequences of HCV genotypes 1a, 1b, 2a and 3a, SARS-CoV-2 and other coronaviruses were derived from previous studies [21,50]. Further alignments of other RNA viruses were constructed for the study from GenBank and the VIPR database [58] using all available or randomly selected sequence subsets as described in S1 Table. Coding region sequences were aligned using MUSCLE [59] as implemented in the SSE package version 1.4 (http://www.virus-evolution.org/Downloads/Software/) [60]. Analysis of viruses encoding single polyproteins (*ie*. picornaviruses, flaviviruses) was based on coding regions only. Regions spanning the start of the first open reading frame (ORF) to the end of the last ORF were used for analysis of viruses with polycistronic genes (coronaviruses, togaviruses, pneumoviruses, filoviruses, hepeviruses and caliciviruses). Alignments are available from the author on request.

### Sequence analysis

Calculation of pairwise distances and nucleotide composition was performed using the SSE package version 1.4. Sequence changes were compiled using the program Sequence Changes with a variability threshold typically set at 5% heterogeneity, where heterogeneity was calculated as the cumulative frequency of all non-consensus bases. Multiple thresholds were used to analyse mutation representation at sites showing different levels of variability (Figs 1 and 2).

Normalised ratios (nC-U) of C->U and U->C transitions (and comparably for G->A and A->G) were calculated as:

nC‐>U=f(C‐>U)/f(U‐>C)*(fU/fC)

where f = frequency.

Normalised 5’ and 3’ context preferences for C->U/U->C transition asymmetries (nC->U) in specific 5’ and 3’ contexts, (5’x)nC->U and (3’x)nC->U (where x represents A, C, G or U) were calculated as:

(f(5’x)nC‐>U/(4xf(x)))/nC‐>U

(f(3’x)nC‐>U/(4xf(x)))/nC‐>U

where f(x) is the global frequency of base x in the sequence.

### RNA secondary structure prediction

Computation of MFE and MFED values was carried out using the program Folding energy scan in the SSE package using sequential 300 base sequence fragments incrementing by 30 bases between fragments. The program call the RNAFold.exe program in the RNAFold package, version 2.4.2 [61] with default parameters.

### Association index calculations

AI values were calculated using the algorithm originally described by Wang *et al*.[52] and Cochrane *et al*. [53] and implemented in the SSE package. An explanation of the method and its underlying algorithm is provided in a Supplementary Methods section (Suppl. Data). The assignment of group labels based on nucleotide identity at sequential sites in an alignment was automated in the program extension, homoplasy scan in the SSE package version 1.5.

### Phylogenetic analysis

Neighbour joining trees were constructed from aligned sequences using the program MEGA7 [62]. Lineage against time plot were derived from data generated in the Phylocom package [27].

### Statistical analysis

All statistical calculations and histogram constructions used SPSS version 26.

## Supporting information

S1 Table Accession numbers of sequences in virus alignments. (DOCX)

S2 Table Compositional features of RNA virus sequence datasets used in the study. (DOCX)

S3 Table Raw and composition normalised frequencies of C->U / U->C transition ratios in different 5’ and 3’ sequence contexts. (XLSX)

S1 Fig Mutation frequencies of RNA viruses. (DOCX)

S2 Fig Unrooted phylogenies of sequence alignments used for homoplasy analysis. (DOCX)

S3 Fig Lineage through time plot for sequence alignments used for homoplasy analysis. (DOCX)
