# Supplementary material for: Extensive C->U transition biases in the genomes of a wide range of mammalian RNA viruses; potential associations with transcriptional mutations, damage- or host-mediated editing of viral RNA
Source: PLoS Pathog. 2021 Jun 1;17(6):e1009596. doi: 10.1371/journal.ppat.1009596 (PMC8195396; doi:10.1371/journal.ppat.1009596)
Supplement: S1 Table — (DOCX) [file ppat.1009596.s001.docx]

TABLE S1

ACCESSION NUMBERS OF SEQUENCES IN VIRUS ALIGNMENTS

**RSV, genotype A VIPR Database Selection of 100**

KM517572, KJ672442, KJ627291, KU316131, KP258740, LC474558, MG642063, KU950502, KX765898, KU950538, KJ627690, MG642032, MN630107, KX655627, KU950474, KU950671, KX765887, KX655692, MH447956, MK167036, KU316137, KU950537, KJ723489, KU950583, MG793382, KU950520, MF001043, MF614946, MG642033, MN630088, KJ672471, MK749909, KP663728, MG642052, KX765934, KU316162, KJ672479, KJ672427, KU950642, KM578843, KJ939957, JX069802, KP258700, JQ901450, KJ672449, KJ627687, KU950652, KJ723474, KX655689, KU950685, KM042390, KJ627273, KJ627287, MG642038, MN306030, KU316168, KX655652, KY883567, KJ939936, KJ672482, KF973330, KU316164, KJ672455, KP258695, KJ939964, KY982516, KJ627733, KX655626, KP258696, KP258704, KP317953, KU950594, MN306054, KX765904, KX655670, MK109776, KJ643531, KX765942, KU950686, KU950501, KJ643465, KJ627343, KU950634, KP258733, KJ627258, KJ627674, MN630102, KJ627719, KF973329, KJ643503, KJ723473, KP258701, KU950541, KJ627723, KU950590, KJ627372, KJ627708, KU950595, KJ627325, KP218910

**BVDV VIPR Database All (no insertions)**

KC853441, MH490942, HV302650, DD122170, AX741975, AF091605, AJ585412, M96751, AJ133739, KY964311, AB078950, MH133206, MH231153, JN704144, KC963967, LC068605, LC068604, LR760748, MH899944, LR699800, LT837585, KP941584, AF220247, LR699799, KR866116, MT079816, MN188073, KF501393, KC695814, KT355592, KC853440, JN400273, MK102095, MH490943, KT896495, LC089875, U86600, KC757383, LC089876, KF772785, KJ620017, KP313732, AF041040, KX987157, LT907992, KX577637, MF166858, MG923683, KP941589, MH899943, EF101530, U63479, KP941588, KY849592, KX857724, DQ088995, KP941581, LT968777, KP941591, KP941590, KP941583, MN188074, KT951841, KT951840, AF526381, KU159365, KT943518, KJ689448, KR029825, JQ799141, KP941592, LT631725, MH899941, MT265675, KF896608, MH166806, KP941587, MH379638, KJ541471, MH899942, MF693403, MK509773, MH899945, KP941586, LT797813, MK509775, KC695810, MK509774, MN623291, KF835699, JX419398, JX419397, KF835697, KF835698, LR699802, LR699801, KU756226, LR699803, MF278651, JQ418633, HQ174296, HQ174295, JN380083, HQ174294, JN644055, HQ174293, JN380080, HQ174292, MF278652, JQ418634, MF172980, JN380089, JN380088, JX297517, JX297521, JX297520, JX297519, JX297518, JX297516, JX297515, JX297514, JX297513, JX297512

**HPeV, genotype 3 GenBank All**

KX068679, KM986843, KJ659490, AB084913, AJ889918, KY020128, JX682576, GQ183029, GQ183033, GQ183032, GQ183030, GQ183031, GQ183026, GQ183027, GQ183028, KY556667, KY556665, KY556659, KY556669, KY556666, KY556664, KY556660, KY556672, KY556662, KY556670, KY556661, KY556668, KY556663, KY556674, KY556675, KY556671, KY556673, MK604044, MK604037, MK604067, MK604040, MK604047, MK604042, MK604039, MK604061, MK604059, MK604062, MF371334, MF371336, MF371333, MF371335, MK604049, MK604046, MK604048, MK604060, KT879915, KT879922, MK604043, KY351616, KY351631, KY351617, KY351618, JX826607, MK604058, MK604052, MK604057, MK604041, MK604045, MK604063, MK604066, MK604038, MK604051, MF797924, MF797928, MF797929, MF797934, KT626009, MF797935, MF797930, KU556748, KU556749, MF797933, MF797926, KY351632, MF797927, MF797925, KU556750, MF797932, MF797916, MF797931, AB668029, AB668030, AB668032, AB759186, AB759187, AB668033, AB668031, LC043117, LC043115, LC043116, LC043114, LC043128, AB759185, AB759191, LC043121, AB759189, LC043127, LC043124, AB759190, LC129275, LC129269, LC129276, LC129272, LC129271, LC129270, LC129273, LC129274, LC383404, LC383392, LC383394, LC383405, LC383407, LC383409, LC383399, LC383408, LC383393, LC383396, LC383400, LC383402, LC383410, LC383391, LC383406, LC383403, LC383401, LC467800, AB759205, AB759204, AB759206, AB759200, AB759201, AB759202, AB759203, AB759199, AB759197, AB759207, AB759192, AB759198, AB759193, AB759195, AB759194

**CHIKV GenBank All**

HM045811, HM045792, HM045810, KX262990, HM045809, HM045823, HM045821, HM045813, HM045786, HM045816, HM045785, HM045788, KY038946, HM045814, HM045805, HM045822, HM045808, HM045815, HM045818, HM045812, KY038947, KX262995, KX262986, HM045791, HM045784, HM045797, HM045800, HM045790, HM045806, KX262988, HM045789, HM045819, HM045820, HM045796, HM045787, KX262987, KX262991, KP702297, KF283986, FR717336, HM045817, JF274082, EF210157, KP003808, KJ941050, HM045794, FJ807896, GQ428211, FJ959103, FJ000068, FJ000067, FJ000066, FJ000065, FJ000064, FJ000063, FJ000062, EU564335, EU564334, KP003809, KP003807, GU189061, GQ428210, KX262996, EU703761, FN295483, FN295484, EU703760, EU703759, GQ428213, GQ428212, KX262993, KX262989, KP003811, KP003810, HM045799, FJ445428, FJ445427, FJ000069, EU244823, EU372006, HM045801, KP003812, KM923918, KM923917, FJ807897, GU199351, FR687343, FR687342, FR687341, FR687340, KJ796851, KJ796849, KJ796848, FN295485, FN295487, JN558835, GU301780, GU013528, GU013529, GU013530, FJ445502, FJ445484, FJ445463, FJ445432, FJ445430, FJ445426, GU199353, GU199352, FJ807898, GQ428215, GQ428214, FJ513675, FJ513673, FJ513657, FJ513654, FJ513645, FJ513637, FJ513635, FJ513632, FJ513629, FJ513628, FJ513679, FJ807899, FJ445511, GU199350, KJ796844, KF151175, KX262997, FR687348, FR687347, FR687346, FR687345, KJ796852, KJ796850, KJ796847, KJ796846, KJ796845, KF151174, JN558836, GU301779, GQ905863, GU908223, KP164869, FR687344, GU301781, KT336777, JN558834, HQ846358, JX088705, HQ846356, KF590567, KF590566, KF590565, KF590564, KC862329, HQ846359, KT336778, JQ861256, JQ861260, JQ861259, JQ861258, JQ861257, KJ679578, KC614648, KJ679577, KP003813, HE806461, KU365292, KT336780, KT336779, KF318729, KC488650, KT308163, KT308161, KT308162, KT308160, KT308159, KT336782, KT336781, KJ579187, KJ579186, KJ579185, KJ579184, KJ689453, KJ689452, AB860301, KJ451623, KJ451622, KM673291, KF872195, KP164570, KP164569, KP164568, KR559482, KR559486, KR559493, KX702402, KR559476, KX702401, KX262994, KX262992, KT192707, LN898111, LN898110, LN898108, LN898107, LN898103, LN898101, LN898100, LN898098, LN898095, KR046234, KR046233, KR046232, KR046230, KR046229, KR046228, KR046227, KR559498, KR559496, KR559495, KR559494, KR559492, KR559490, KR559488, KR559487, KR559485, KR559484, KR559483, KR559481, KR559475, KR559474, KR559470, KR264951, KR264949, KP164571, KP164567, KP851710, KP851709, KR264950, KU940225, KR559473, KY057363, KX228391, KX496989

**EBOV GenBank Selected**

AF086833, KC242801, KY425630, KY425637, KY425639, KY425647, KY425649, KY425652, KY425656, MH121166, MH121168, KR063671, KC242791, KC242792, KR063672, JQ352763, KR867676, KU182898, KU182899, KU182900, KU182901, KU182902, KU182903, KU182904, KU182905, KU182906, KU182907, KU182908, KU182909, KY425636, KY425653, MG572235, KC242796, KC242799, KR824526, MH121165, KC242793, KC242794, KY785939, KY785940, KY785947, KY785948, KY785949, KY785965, KY785969, KY785970, KY786017, KC242800, KF113528, KC242784, KC242785, KC242786, KC242787, KC242788, KC242789, KC242790, HQ613403, MH733477, MH733478, MH898466, MH733479, MH733480, MH733481, MH733482, MH733483, MH733484, MH733485, MH733486, MH733487, MH733488, MH733489, MH733490, MH733491, MK007329, MK007330, MK163644, MK163645, MK163646, MK163647, MK163648, MK163649, MK163650, MK007331, MK007332, MK007333, MK007334, MK007335, MK007336, MK163651, MK163652, MK163653, MK163654, MK163655, MK163656, MK163658, MK163660, MK731985, MK731987, MK731988, MK731993, MK007337, MK007338, MK007339, MK007340, MK007341, MK007342, MK007343, MK007344, MK731986, MK731989, MK731990, MK731994, MK163661, MK731992, MK163662, MK163663, MK163665, MK163666, MK163667, MK163668, MK163669, MK163670, MK163672, MK163673, MK163674, MK163675, KU143777, KT725268, KX009901, MF599519, KT725257, KR105295, KR653240, KR534585, KP759733, KP759660, KT725311, KR817226, KR817112, KM233085, KR534564, MH425138, KY426709, KR075003, KR105300, KR817224, KP759692, KY401669, KP184503, KR534517, KT357815, KR817200, KU143821, KY558986, KU143778, KT725380, KX121424, KR653304, KR817126, KM233088, KU143812, KR105249, KY471125, KR817222, KM233106, KM233058, KX009897, LC152433, AY354458, KP759674, KU052670, KR534528, KP759724, KM233116, KR653279, KT357841, KP759728, KR105323, KM233050, KR653301, KY007525, KT725296, KR817082, KU143833, KR105316, KR105244, KR534574, KR105283, KY426704, KY471092, KR534546, KP728283, KR534552, KT725349, KU296527, KM233087, KT013257, KR817111, KT725319, KR105232

**MeV GenBank All**

AF266288, AY730614, JF727649, AY486083, AF266290, AF266289, AB591381, AF266287, AF266286, Z66517, EF033071, AB046218, FJ416067, S58435, MN145872, K01711, EU435017, DQ345721, MG912589, AB016162, AB032167, DQ227321, JF791787, LC336599, EU293548, EU293552, EU293551, DQ227319, EU293549, AB481087, AB012948, AB481088, AB012949, DQ227318, JN635404, JN635407, DQ227320, MN131123, MH356254, JN635402, MH356236, KY969480, MN125020, HM439386, MG912590, JN635403, JN635410, MH356241, MH356250, MF496202, MF449469, KC164757, MN125028, MH356253, MN131117, MF496200, MH356244, MH631015, MH356239, MF496201, MH631016, MH356243, MG912593, MH356242, GQ376026, MK513625, MH356240, MH638233, GQ376027, MH173047, JN635405, MH356245, JN635406, KJ410048, MK161348, MG912592, JN635408, KY969479, MH356248, KJ018971, MH356237, AB254456, KU728742, JN635409, KU728743, MT738550, MH356252, MK142916, MK142914, MK142915, MH356238, MH356246, KT732231, MH356247, KC117298, MH356255, MH356249, MT789844, MT789833, MT789849, MT789848, MT789839, MT789838, MH356251, MT789842, MT789840, MT789832, MT789824, MT789827, MT789821, MT789847, MT789829, MT789825, MT789823, MT789836, MT789830, MT789837, MT789826, KY969477, MT789828, MT789835, MT789843, MT789834, MT789822, MT789831, KT732230, KY656518, KT732225, MT789845, KT732258, KT732254, MT789841, KT732244, KT732237, KT732234, KT732233, KT732227, KT732226, KT732256, KT732246, KT732240, MT789846, KT732235, KT732228, KT732241, KT732251, KT732232, KT732229, KT732249, KT732247, KT732243, KT732252, KT732255, KT732245, KT732260, KT732259, KY969476, KT732261, KX838946, MN630022, KY969478, MG912591, KT732214, KY969481, MN017369, MN893225, KC164758, MN630023, KT732219, KT732224, KT732222, KT732221, KT732217, KT732223, KT732216, KT732215, MF775733, KT732220, KT732218, MT789797, MT789815, MT789818, MT789805, MT789804, MT789800, MT789799, MT789795, MT789793, MT789792, MT789813, MT789796, MT789814, MT789803, MT789802, MT789801, MT789798, MT789790, MT789789, MT789788, MT789807, MT789806, MT789816, MT789812, MT789811, MT789791, MT789808, MT789817, MT789810, MT789819, MT789820, MT789794, MT789809, MK513610, MK513616, MG912594, KJ755975, FJ161211, DQ211902, JN635411, KJ018970, MN630021, MN630020, MG972194, KT588921

**EV-A71 GenBank All**

MK652139, DQ341367, MG976581, KX372324, JX244184, MH716347, JQ639384, LT719065, LC506514, KC436271, MG214681, KT428646, KP289432, MH716380, JF738002, HQ647173, GQ279369, KU936120, HM807310, FJ607337, FJ357384, MG367595, LC321989, MH716337, AB575917, KX372322, KJ004560, KF982854, JQ074189, MG367594, EU131776, LT719063, LT719067, AB575918, MN254979, MF662695, KJ400360, U22522, KJ686246, KU936132, AB575938, HM002487, HQ647172, KJ686249, JN992284, KC436265, AB575935, KU936128, FJ194964, KJ784495, AB747375, JX678885, DQ341358, LC375764, AB575928, MF973167, LC506513, FJ357376, LK985324, MG672479, KF142413, MG773126, KX372317, KP289419, KP289425, KP289422, AB575911, KT345959, MG672481, GQ994992, DQ341364, DQ341357, KU936121, KC436266, KY612315, KC109780, AB575914, GQ231939, MH111073, JQ681218, KJ686308, MH716310, AF302996, GQ994989, KY315729, AM396584, LT719064, KM055005, KU254596, KP308450, HQ456306, KC436270, MG367600, KU936125, JQ742001, KC954664, FJ607336, HQ189392, KT008669, DQ341355, JQ742002, KX372328, JQ280307, KX372310, KM673244, DQ341359, KT428647, AM396587, KJ686262, FJ606447, KP289431, KX197462, AB575936, MG672478, LT719068, KP289424, EF063152, MG207962, EU414333, FJ360545, AF316321, JF738001, MF662701, KJ686208, KP308426, JF799986, MH484070, KF974790, GQ231934, U22521, AB575923, GQ994991, MG431943, JQ316638, DQ341360, KX372308, AY465356, HQ647179, LC506515, LC506516, AB204852, KF501389, AB204853, GU434678, MH484071, MH484069, AB482183, MH484068, AB747373, MH484067, MH484066, KX139462, MG432108, KF514878, AJ586873, GQ231936, FJ357385, GQ231942, KT354870, KT354867, KF974789, LC321990, GQ231935, KX372330, KT354869, KT354868, KT354866, KF974784, MH716379, KF974779, GQ231943, GQ231925, MG756712, KT354872, KT354871, MG756729, KU641504, KU641502, GQ231941, KT354875, KT354874, MH716377, MG756708, KX372314, KU641505, KX372327, KF154355, MG756743, MG756709, AB575913, MG756713, LC375765, KF974794, AB550337, MG756714, KU641503, KF974781, KF974797, KF974792, MG756749, KU641507, KU641501, MH716376, MH716386, KU641506, KF974780, MG756710, MH716384, KX372316, MH716348, KX372323, AB575927, MH716391, MG756734, KF974785, KF974783, MH716389, MG756740, FJ357375, MH716383, MG756750, MG367599, MG756742, MG756733, MG756727, MG756723, MG756721, KX372331, MG756752, HQ647171, MG756746, KF974787, MG756741, MH716390, KU641508, MH716353, MH716351, MH716300, MH716299, MG756744, MG756730, MG756737, KF974796, KF974791, MH716361, MG756747, MH716350, MG367598, AM396585, MG756753, KY074644, MH716362, MH716349, KP691653, MH716360, MG756736, MG756735, MG756745, FJ357382, MH716319, MG756726, MH716364, KP308454, KF974793, MH716292, MG756751, MG756706, FJ357380, KJ686176, KF974795, MH716385, KP691659, KJ686137, MH716366, MG756728, JX678878, MG756711, AB550333, MG756732, JX025561, MH716288, MH716286, MG756748, KX372326, JX678886, DQ341362, MH716388, KX430824, MH716346, MG756738, KM508794, KJ686302, KJ686131, MH716387, KP691660, GQ231930, GQ231928, EU527985, MN629889, MH716287, MH716332, MH716313, MH716304, KJ686225, MH716393, MH716311, AB550338, FJ607335, MH716352, KJ686270, GQ231929, GQ231927, MH716267, EU753365, MH716305, MH716285, AB550339, MH716284, JX678882, KJ686128, GQ279370, MH716298, MH716259, MH716290, MH716282, MH716281, KP691643, MH716344, JQ965759, KF974798, KF154354, MH716315, EU753407, MH716291, MH716272, MH716271, MH716373, MH716274, KJ686211, KJ686264, AF176044, MH716318, MH716317, MH716314, KP691657, MH716334, MH716270, MH716293, MH716278, KP691663, JQ517316, FJ357374, KF514880, MG756731, KX372311, JN544418, EU753375, HM622390, AF304458, MH716283, KP691648, MH716381, MH716333, MH716265, FJ713137, MH716260, MH716356, MH716268, MH716266, AF352027, MH716280, KJ686222, MH716308, MH716369, KY952186, KR045298, EU753398, MH716269, KX372320, KP691654, JQ708210, JN020147, MH716273, HQ611148, MG756739, KJ686140, AF136379, MH716277, KP691647, MH716302, MH716297, KP691658, MH716374, MH716336, MH716258, KJ686192, MH716316, KX372313, KP691664, JN992282, JN544419, MH716303, KP691652, GQ994988, KJ686297, MH716261, MH716309, GQ231926, MH716279, KP691651, MH716331, KP691650, AB550341, MH716342, GQ231940, MK904809, KP691655, AF304459, MH716324, MH716294, GQ231937, GQ231932, DQ341365, JQ724182, JQ074188, KF134486, MH716275, GQ231931, MH716358, MG756704, JN864018, MH716323, MH716276, KP691662, MH716355, MH716327, MG756698, KU936130, KX197459, JQ074187, KP308430, JN992285, MF662687, MH716296, JN256063, KP308419, KJ686234, AB469182, JX678877, JX678876, AB550334, EU703813, JN256064, AB550335, KM077140, EU376004, MH716325, KJ004556, HM053669, MH716328, KJ746494, KX197457, JX678883, DQ341368, MG756696, EU414334, JX986739, HQ891924, FJ606448, EU703814, KX197463, KP308449, MH716329, MH716252, EU376005, EU364841, MG756702, KY074643, KP691645, JF830007, GU396280, FJ158601, MH716335, KC436269, HQ891923, EU864507, MH716257, KC954663, DQ060149, FJ606450, EU414335, EU703812, DQ381846, MH716354, KP691665, KY425527, KJ686195, HQ456311, FJ194965, MH716321, MH716256, MH716341, KP691656, FJ158600, MH716370, JX025559, FJ607338, KR045302, KP308459, KP308428, KJ686226, KU574619, KR045301, JX678884, JX678880, JX678875, MF662686, HQ456305, AM396586, JN256060, HM002489, KP308434, JX111890, AM396588, JQ708209, JX111892, HM002485, AF119796, MG756725, KX372318, JX111888, MH716248, MG756697, MG756700, JX678881, HQ694982, MG756699, KJ686237, KJ686198, EU414331, MF662685, MH716254, KJ686269, KJ686200, KJ686194, KJ686157, MF662681, KY014080, KJ686184, JN256059, HQ891927, JX244183, KP308457, KP308401, GU459071, KP308409, KJ686307, KJ686261, KX752783, KP308453, KP308441, KJ686204, KJ686151, HM622392, MH716255, KP308408, KJ686280, KJ686256, KJ686248, KJ686166, HQ456310, KJ686224, KJ686223, JF913464, KX372332, KX197458, KP308442, KP308436, KJ686238, MF662684, MH716340, MH716253, KP308439, KJ686206, JX111891, MH716251, KP308407, JX244186, FJ439769, MG756722, HQ694985, KP308437, KJ686245, KJ686168, KJ686142, KJ686127, JX244185, HQ891928, KP308432, KJ686266, KJ686217, KJ686201, KJ686165, KJ686295, FJ360546, KP308415, GU459070, KP308460, KP308440, KJ686258, KJ686182, KJ686162, KJ686143, JX111893, JX017384, KT008671, KP308412, KJ686299, MH716250, KP308422, KP308402, KJ686284, KJ686252, KJ686251, KJ686163, KJ686152, HQ891925, FJ600325, KJ686268, KJ686185, KF826491, KT008672, KJ686193, KJ686305, KJ686229, KJ686239, KJ686236, KJ686170, KJ686156, KJ686149, KJ686141, KJ686279, KJ686216, HQ694983, KP308452, KP308438, KJ686241, HM622391, KP308403, KJ686303, KJ686283, KJ686267, KJ686263, KJ686231, KJ686213, KJ686210, KJ686155, KJ686132, KJ686130, KJ686221, KJ686188, KJ686287, KJ686174, KJ004559, KJ686286, HQ647177, KP308433, KJ686214, KJ686202, KJ686161, KJ686160, KR045300, KJ686175, EU414332, HQ882182, HQ694986, HQ694984, HM003207, MK028135, HQ647178, KJ686196, KJ686172, MF662680, KJ686289, MG773125, KY014079, KT008670, KJ686197, KJ686153, KP308423, KJ686259, KJ686171, KP308451, GU198368, KT428649, KJ686300, MF662678, KP274877, KJ686298, KJ686181, KJ686158, GU198369, KJ686189, KJ686129, GU198367, JF894383, JF894381, KJ186973, KJ686243, KJ686186, KJ686154, HQ129932, KJ686257, KJ686255, KJ686228, KR045297, DQ341356, JN864022, KJ686169, MG756718, HQ647167, KY014078, KJ686288, KJ686271, KJ686227, KJ686207, MF662683, KJ686275, MG756717, MG756715, KJ686291, KJ686242, JF894382, KJ686282, KJ686281, KJ686276, KJ686235, KJ686215, KJ686205, KJ686150, KJ686145, KP308431, MG756716, KT428650, KJ686164, KJ686253, KJ686146, HQ647174, KJ686240, KJ686233, KJ686183, KJ686139, HQ647176, KJ686285, MF662699, KJ686173, KJ686136, MF662700, MG976582, KJ686209, HQ647168, MG756691, KJ686167, KJ686138, KU936131, KJ686304, KJ686190, MG756692, LC321992, KP308414, KJ686301, KP308458, KJ686247, KR045304, KJ686294, KJ686232, KR045293, KP308425, GU198371, KP308429, KJ686260, GU198370, KJ686278, KJ686212, KX197455, KP308418, KJ686293, KR045292, KR045291, KP289426, KP289420, KP308445, KX197461, KP308424, KM211579, KU936129, KP308421, KT428644, KR045294, KX197456, KR045295, MF405075, KT428648, MG756719, JF820315, KP308446, HQ647169, KP308444, JF820312, HQ424434, HQ424435, KC296445, KP274876, JF820316, JF820313, MG773124, MG207963, FJ172159, HM752814, JF820314, AB575941, KC296443, HQ423143, HQ424433, KX430820, KP308406, KX430823, KP274874, KP274875, KP308417, KR045303, KX430819, KM593929, KJ686187, KJ686144, KJ686191, KJ686179, KJ686230, AF119795, EU527983, KP308456, KP308411, KJ632499, KP308416, KP308448, KX430818, LC321993, MH718269, KP308427, KF543271, KX197465, KP308404, KP308447, KP308420, KF306100, HM156065, KU159440, KX588236, KU159436, KP308435, KJ186974, KF306101, HQ850973, JN230523, KU159435, KU159441, MH511208, MH511205, MH511194, MH511182, MH511207, MH511202, MH511198, MH511197, MH511196, MH511195, MH511191, KU159442, MH511204, MH511192, MH511187, MH511185, MH511184, MH511181, KU159439, MH511206, MH511203, MH511201, MH511200, MH511199, MH511190, MH511188, MH511186, GU350629, MH511193, JQ950555, KU159437, KU159438, KJ686134, KU159434, KX430817, AB747374, MG013988, MG672480, MK800119, KF974786, AB575916, LC321991, FJ357379, FJ357383, MH716378, KX372315, KF974782, MG367596, KT354873, FJ357381, AB550336, KY888026, KF974788, KF514879, MG756695, MH716263, FJ357378, MG756707, JN964686, MH716392, DQ341363, MH716262, KJ686296, KX372329, MH716382, MH716307, MG756754, MH716359, MH716363, MH716306, KP266579, MG756694, MH716312, AB550332, KP691644, MH716365, KP691649, KJ746493, MH716289, MH716301, FJ607334, MH716371, JX678874, DQ341366, KX372325, KU647000, KX372312, DQ452074, LC375766, JN052925, KC436267, FJ357377, MH716343, KP691661, KC954662, MH716372, AF304457, MH716368, MH716345, DQ341361, EF373575, EU753397, GQ231933, FJ360544, HQ456309, HM053670, MH716320, MH716357, MH716322, AB550340, KX372321, GQ231938, AB575915, MH716295, FJ357373, MF662682, GQ994990, MH716367, KF142412, EU753384, MH716375, MH716326, JN864020, JX244182, KF312457, MH716338, JQ319054, GU366191, DQ133459, JN256062, KP691646, FJ461781, MH716330, MH373639, MG208882, JN992283, HM002484, MH716264, JN001860, KC436268, KP266572, JQ086365, KJ004558, KF444809, KX197464, DQ133458, HQ456312, AB575912, FJ606449, JN256061, MH716339, JQ806378, KP289421, HQ456308, MH716249, MF662688, KC436272, JQ514785, MG756701, MG756703, MK344771, KJ686254, KX372319, HQ998852, DQ341354, KP308443, JX111889, HM002488, GQ892830, HQ456307, MF662679, MG756724, JX244187, JN864019, KJ004557, KJ686292, HQ825317, HQ456313, KC414134, HQ400942, KJ686244, JQ804832, JX986737, HQ407557, JF738000, KM402021, KJ686265, KM591215, HQ325852, HQ188292, KJ004554, MF662690, KP198623, KJ686272, KP198624, KJ784496, KJ004555, JQ074190, EF373576, MG212485, JQ736684, JQ086366, KJ686218, MF662693, KT428645, HQ423142, MG773123, KX372309, MG756720, KJ004552, HQ828086, KP289428, HM245927, MF362981, KU936124, KP289417, MF662677, KJ004553, MF662697, MF662689, HQ712020, KX197460, KJ686220, KC570452, HQ891929, HQ426649, MF662691, KJ686177, KJ686219, MF662696, KP289423, KJ686148, KP308405, KP861243, JN864023, MF662694, HQ647175, HQ891926, MK307505, KU936123, KJ686199, MF662692, KJ686203, KJ686147, KJ508182, HM245928, KJ686133, AB575937, KU254595, KC570453, HQ647170, FJ828519, KJ686290, KP289429, MG756693, KJ686274, KJ686273, KT345960, KP289430, KF668443, KU936126, MG875331, KP289427, KJ686180, KY582572, KJ686250, KM211580, MG520666, KU936127, JN835312, MF431793, KU936122, KR045296, KJ686135, MG581490, HQ424436, JQ639383, KF142411, EU812515, MG773122, KP289418, MF662698, AB575942, AB575948, HQ424437, AB575939, MG756705, KX893543, GU196833, KY888025, KJ632497, KJ686178, KJ632498, MH511183, MH511189

**Porcine KoV GenBank All**

MF506730, AB624493, LC210613, LT898428, GQ249161, EU787450, LC210612, LC210606, LC210600, LC210601, LC210603, LC210602, LC210610, LC210604, LC210620, LC210599, JQ692069, MG800803, MG800804, MG800807, MG800806, LC210611, LC210622, MF062451, MF062445, MF062449, LC210618, KC424640, KC424638, KC424639, KY234500, GU292559, LC210617, LC210616, MK962328, MK962332, MK962335, MK962336, MK962321, MK962330, MK962320, MK962333, MK962334, MK962323, MK962327, MK962329, MK962331, MK962324, JX177612, MK962322, MK962326, MK962325, LC210608, LC210609, LC210605, LC210607, LC210619, LC210621, KM977675, KP144318, KP260507, KT892971, LC210614, LC210615, JN630514, JX827598, MF062440, KJ452348, MF062448, MF062438, MF062437, KC204684, MG800805, JX401523, KM051987, MF062436, KC414936, KF539763, KF695124, MF062447, MF062443, MF062444, MF062450, MF062452, MF062441, MF062442, MF062446, MF062439, KY234499, MF062435

**DENV1 GenBank All**

AB608788, AB608786, AB608787, LC016760, JX669468, KF971870, KF971871, GU131922, EU848545, KC172829, AF298808, FJ196847, KF955441, KF921948, AF514889, EU482567, AF180817, JN903580, JQ922544, GU131887, AY277666, AY722802, AY708047, JQ922545, denv, LC011948, AB189121, AB195673, AB204803, KF921911, KC762631, KC762652, AB074760, JX669463, JX669465, HM469967, JN903579, FJ850070, GU131956, KF955442, GU131966, KJ189349, KJ189350, FJ687426, FJ205875, FJ410188, FJ850099, DQ285560, EU081257, KF955406, AY732483, KP723476, AF350498, KF672763, FJ196846, AB178040, KJ468234, HE795086, LC011945, LC011949, HM469966, JX669462, JX669464, JX669466, JX669467, JX669469, JX669470, JX669471, JX669472, JX669473, JX669474, JX669475, KF971869, JN903578, JN903581, KJ649286, KF887994, FJ850073, FJ850075, FJ850077, FJ850081, FJ850084, FJ850087, FJ850090, FJ850093, GU131863, GQ868559, GQ868560, GQ868561, GU131948, GU131949, GU131888, GU131889, GU131890, GQ868630, GU131891, GU131892, GU131893, GU131894, GU131895, GQ868632, GQ868633, GU131919, GU131920, GU131921, GQ868635, GQ868636, GU131923, GQ868637, GQ868639, GU131925, GU131926, FJ639669, FJ639670, FJ639671, JN819423, FJ639672, FJ639673, FJ639674, FJ639675, FJ639676, FJ639677, GQ868618, GQ868619, FJ639678, FJ639679, FJ850069, FJ639680, FJ639682, JQ287664, FJ639683, FJ639684, FJ639685, FJ639686, FJ744702, FJ639688, FJ639689, FJ639690, FJ639691, FJ639692, FJ639693, FJ639694, FJ639695, FJ639696, HM631852, KF955444, HM181936, HM181937, HM181938, HM181939, HM181940, HM181941, HM181942, HM181943, HM488255, HM181944, HM181946, JQ287665, HM181948, HM181949, HM181950, HM181951, HM181952, HM181953, KF921933, HM181954, HM181955, HM181958, HM181959, JF937651, KF955415, FJ687428, FJ687429, FJ850068, FJ687430, FJ687432, FJ687433, JQ922547, EU660390, EU660391, EU660392, EU660393, EU660394, EU660401, FJ373305, EU660402, EU660403, EU660397, EU687247, EU660395, EU660412, EU660418, EU677150, EU677151, EU677152, EU677153, EU677154, EU677155, EU677156, EU677157, EU726777, EU677158, EU677159, EU677160, EU677161, EU677162, EU677163, EU677164, EU677165, EU677139, EU677140, EU677166, EU677167, EU677168, EU677170, EU726778, EU677171, EU677172, EU677173, EU677174, EU726779, EU660419, EU677175, EU677176, EU726780, EU726781, EU677177, EU726782, EU677178, EU687251, FJ024426, FJ024427, FJ024428, FJ024429, FJ024430, FJ024431, FJ024432, FJ024433, FJ024434, FJ024435, FJ024436, FJ024437, FJ024472, FJ024439, FJ024441, FJ024442, FJ024443, FJ024444, FJ373296, FJ182018, FJ410287, FJ182019, FJ432719, FJ182020, FJ024445, FJ182021, FJ182022, FJ024446, FJ182023, FJ024447, FJ182025, FJ373297, FJ024448, FJ182026, FJ373298, FJ182027, FJ390381, FJ182028, FJ024449, FJ390382, FJ182003, FJ182029, FJ390383, FJ024450, FJ182030, FJ182031, FJ182033, FJ182034, FJ182036, FJ410289, FJ205876, FJ024455, FJ024456, FJ024457, FJ390386, FJ024459, FJ205881, FJ024460, FJ205882, FJ205883, FJ024462, FJ024463, FJ390388, FJ024464, FJ410191, FJ432723, FJ410192, FJ410194, FJ432725, FJ432727, FJ432729, FJ432730, FJ432732, FJ432733, FJ410196, FJ410197, FJ432734, FJ432735, FJ432736, FJ410198, FJ432737, FJ410199, FJ432738, FJ432739, FJ432740, FJ410201, FJ410203, FJ410204, FJ432742, FJ410205, FJ410206, FJ410207, FJ432744, FJ547060, FJ432745, FJ410209, FJ410210, FJ432746, FJ410211, FJ432747, FJ432748, FJ432749, FJ461306, FJ410212, FJ461307, FJ410213, FJ461308, FJ461310, FJ410216, FJ461312, FJ906728, FJ461313, FJ547063, FJ410218, FJ461315, FJ461316, FJ461317, FJ410220, JF937649, FJ410222, FJ461318, FJ461319, FJ461320, FJ410225, FJ410226, FJ410227, FJ859029, FJ547065, FJ410230, FJ410231, FJ461323, FJ461324, FJ461325, FJ461327, FJ461328, FJ410234, FJ410235, FJ410236, FJ410238, FJ410239, FJ410240, FJ410242, FJ410243, FJ410244, FJ410245, FJ461330, FJ410246, FJ410247, FJ410248, FJ410249, FJ410250, FJ410251, FJ410252, FJ410254, FJ461331, FJ410255, FJ410256, FJ410257, FJ461332, FJ410258, FJ461333, FJ410260, JF937650, FJ410261, FJ461335, FJ410262, FJ410263, FJ410264, FJ410265, FJ461336, FJ410266, FJ410267, FJ410268, FJ410269, FJ410270, FJ410272, FJ410273, FJ410274, FJ410275, FJ410276, FJ410277, FJ410278, JQ287667, FJ410279, FJ410280, FJ410281, FJ410282, FJ410283, FJ410284, FJ410285, FJ410286, FJ461339, FJ461340, FJ461341, FJ882515, FJ882516, FJ882517, GQ199771, KF921934, JQ287660, JQ287661, FJ882518, FJ882519, FJ882520, FJ882521, FJ882522, FJ882523, FJ882524, FJ882525, FJ882526, FJ882527, FJ882528, FJ882529, FJ882530, FJ882531, FJ882532, GQ868605, FJ882533, FJ882534, FJ882535, FJ882536, FJ882537, GQ868606, FJ882538, FJ882539, FJ882541, GQ199772, FJ882542, FJ882543, FJ882544, FJ898371, FJ882545, FJ898372, FJ898373, FJ898374, FJ898375, FJ898376, FJ898377, FJ898378, FJ898379, GQ199773, GQ868607, JF937596, FJ906963, FJ906964, FJ906965, FJ898380, FJ898381, FJ898382, FJ898383, FJ898384, FJ898385, GQ868608, GQ199774, GQ199775, JQ287662, FJ882546, KF921935, GQ199776, GQ199777, JQ287663, GQ199778, GQ199779, GQ199780, GQ199781, GQ199782, GQ199783, GQ199784, GQ199785, GQ199786, GQ199787, GQ199788, GQ199789, GQ199790, GQ199791, GQ199792, GQ199793, GQ199794, GQ199795, FJ882547, GQ199796, GQ199797, GQ199798, GQ199799, GQ199800, FJ882548, GQ199801, GQ199802, JF937597, GQ868609, GQ199803, GQ199804, GQ199805, GQ199806, GQ199807, GQ199808, GQ199809, GQ199810, GQ199811, GQ199812, GQ199813, FJ882549, GQ199814, GQ199815, GQ199816, GQ199817, GQ199818, FJ882550, FJ882551, GQ199819, FJ882552, FJ882553, FJ882554, GQ868610, GQ868611, GQ199820, FJ882555, GQ199821, FJ882556, GQ199822, FJ882557, FJ882558, GQ199823, GQ199824, FJ882559, FJ882560, GQ199826, GQ199827, GQ199828, FJ882561, GQ199829, FJ882562, FJ882563, FJ882564, FJ882565, GQ199830, FJ882566, FJ882567, GQ199831, GQ199832, FJ882568, FJ882569, GQ199833, FJ882570, GQ199834, GQ199835, FJ898386, GQ199836, GQ199837, FJ898387, FJ898388, FJ898389, FJ898390, FJ898391, FJ898392, FJ898393, FJ898394, FJ898395, GQ199838, FJ898396, GQ199839, FJ898397, FJ898398, FJ898399, FJ898400, FJ898401, FJ898402, FJ898403, FJ898404, GQ199840, FJ898405, FJ898406, GQ199841, GQ199842, FJ898407, FJ898408, GQ199843, FJ898409, GQ199844, GQ199845, JF937598, GQ199846, GQ199847, GQ199849, GQ199850, FJ898410, GQ199851, FJ898412, GQ199852, FJ898413, FJ898414, FJ898415, GQ199853, GQ199854, FJ898416, FJ898417, GQ868612, GQ199855, FJ898418, FJ898419, FJ898420, FJ898421, FJ898422, FJ898423, GQ868613, FJ898425, FJ898426, GQ199856, FJ898427, FJ898428, FJ898429, JF937599, FJ898431, GU131678, GU131679, GU131680, GU131681, GU131682, GU131683, GU131684, GU131685, GQ868614, GU131686, GU131687, GU131688, GU131689, GU131690, GU131691, GU131692, GU131693, GU131694, GU131695, GU131696, GU131697, GU131698, GU131700, GU131701, GU131702, GU131703, GU131704, GU131705, GU131706, GU131708, GU131709, GU131710, GU131711, GU131712, GU131713, GU131714, GU131715, GU131716, GU131717, GU131718, GU131719, GU131720, HM181960, HM181961, HM181963, HM181964, HM181965, HM181966, GU131721, GU131722, GU131723, GU131724, GU131725, GU131726, GU131727, HM631850, GU131728, KF955446, KF921942, JF937602, GU131729, GU131730, GU131731, GU131732, GU131733, GU131734, GU131735, GU131736, GU131737, JF937603, GU131738, JN000935, JF937604, JF937605, JF937606, JF937607, JF937608, GU131739, GU131740, GU131741, GU131742, GU131743, GU131744, GU131745, JN093516, GU131746, JF937609, KF921949, GU131747, GU131748, GQ868615, GU131749, GU131750, GU131751, GU131752, GU131753, GU131754, GU131755, GU131756, GU131757, GU131758, GU131759, GU131760, GU131761, GU131762, GU131763, GU131764, GU131765, GU131766, JF937610, GU131767, HM181967, JF937611, GU131768, GU131769, GU131770, GU131771, GU131772, GU131773, GU131774, GU131775, GU131776, GU131777, GU131778, GU131779, GU131780, GU131781, GU131782, GU131783, GU131784, GU131785, JF937612, GU131786, GU131787, GU131788, HM181968, GU131789, GU131790, HM181969, GU131791, GU131792, HM488256, GU131793, GU131794, GU131795, GU131796, GU131797, GU131798, GU131799, GU131800, GU131801, JF937613, JF937614, GU131802, GU131803, GU131804, GU131805, HM631851, GU131806, GU131807, GU131808, GU131809, GU131810, GU131811, JF937615, GU131812, GU131813, GU131814, GU131815, GU131816, GU131817, GU131818, GU131819, GU131820, HM181970, GU131821, GU131822, GU131823, GU131824, GU131825, GU131826, JF937616, JF937617, JF937618, JF937619, GU131827, GU131828, GU131829, GU131830, GU131831, EU482789, EU482790, EU482791, EU482792, EU482706, EU482707, EU482708, EU482709, EU482710, EU482711, EU482712, EU482713, EU482714, EU482715, EU482717, EU482718, EU249490, EU249491, EU249492, EU249493, EU249494, EU249495, EU482793, EU482795, EU482796, EU482797, EU482798, EU482799, EU482800, EU482802, EU482803, EU482804, EU482805, EU482806, EU482807, EU482808, EU482809, EU482810, EU482811, EU482812, EU482813, EU482814, EU482815, EU482816, EU482817, EU482818, EU482819, EU482820, EU482821, EU482822, EU482823, EU482824, EU482825, EU482826, EU482827, EU482828, EU482476, EU482477, EU482478, EU482479, EU482481, EU482482, EU482483, EU482484, EU482485, EU482486, EU482487, EU482488, EU482489, EU482490, EU482491, EU482492, EU482493, EU482494, EU482495, EU482496, EU482497, EU482498, EU482499, EU482500, EU482501, EU482502, EU482504, EU482505, EU482506, EU482508, EU482509, EU482510, EU482511, EU482512, EU482513, EU482514, EU482515, EU482516, EU482517, EU482519, EU482520, EU482521, EU482522, EU482523, EU482524, EU482525, EU482526, EU482527, EU482528, EU482532, EU482533, EU482539, EU482540, JQ045626, JQ045628, JQ045629, JQ045630, JQ045631, JQ045632, JQ045633, JQ045634, JQ045636, JQ045635, JQ045637, JQ045638, JQ045639, JQ045640, JQ045641, JQ045645, JQ045642, JQ045644, JQ045646, JQ045647, JQ045648, JQ045649, JQ045650, JQ045651, JQ045652, JQ045653, JQ045654, JQ045656, JQ045657, JQ045658, JQ045659, JQ045660, JQ045661, JQ045662, JQ045663, JQ045664, JQ045665, JQ045666, JQ045667, HQ624983, FJ384655, JQ048541, JN697056, JN697057, HQ624984, KP398852, HQ891313, HQ891314, HQ891315, HQ891316, JN054256, JN054255, KJ438293, KJ438296, KC692495, KC762625, KC762654, KC762636, KC762635, KC762650, KC762623, KC762632, KC762646, KC762637, KC762620, KC762621, KC762643, KC762651, KC762638, KC762640, KC762628, KC762644, KC762630, KC762641, KC762648, KC762622, KC762634, KC762633, KC762629, KC762627, KC762647, KC762639, KC762642, JQ915077, GU370049, HM469968, KC172834, KJ933413, EU359008, AY835999, KF672760, FJ176780, GQ398255, JN205310, HQ332182, KP406803, KP406801, KC131140, KT279761, KP723473, KJ726663, KJ726662, KJ189316, HM631855, JN638344, JN638343, JN638342, JN638341, JN638340, JN638339, JN638338, JN638337, JN638336, FJ469909, FJ469908, FJ469907, AB519681, EF025110, AF226685, AY145123, EU081254, EU081281, EU081280, EU081279, EU081278, EU081277, EU081276, EU081275, EU081274, EU081273, EU081272, EU081271, EU081270, EU081268, EU081267, EU081266, EU081265, EU081264, EU081263, EU081260, EU081259, EU081258, EU081256, EU081255, EU081253, EU081252, EU081251, EU081249, EU081248, EU081247, EU081246, EU081245, EU081244, EU081243, EU081241, EU081239, EU081238, EU081237, EU081236, EU081235, EU081234, EU081233, EU081232, EU081231, EU081230, EU081229, EU081228, EU081227, EU081226, EF457905, AY732482, AY732481, AY732480, AY732479, AY732478, AY732477, AY732476, AY732474, HG316481, U88537, AB074761, AF298807, AY726555, AY726554, AY726553, AY726552, AY726551, AY726550, AY726549, AY722803, AY713475, AY713474, AY713473, KF864667, JQ915078, FJ196843, FJ196842, KJ726665, KF921951, KF921943, KF921940, KF921939, KF921936, KF921932, JQ692085, JQ917404, GU370048, DQ285558, KP686070, KJ726664, AY732475, AY762084, EU179861, AY722801, AY713476, AB189120, GQ868602, JN697058, KC762653, KC762645, KC762624, KC762626, JQ915079, JQ915080, JQ915072, JQ915071, JQ915073, JQ915074, JQ915075, JQ915076, KF289072, KC172835, FJ176779, KM204119, DQ285562, DQ285559, AF311956, KP406802, DQ672563, DQ672562, DQ672560, DQ672559, DQ672558, DQ672557, DQ672556, AF309641, JQ045627, AY145122, AY145121, AF226687, EF032590, HG316482, U88535, AF226686, FJ196848, FJ196845, FJ196844, FJ196841, KR028435, JX669461, KF184975, JQ675358, FJ850071, GQ868562, GQ868563, GQ868564, GQ868565, GQ868566, GQ868567, GQ868568, GQ868569, GQ868570, KJ189302, KJ189303, KJ189304, JQ922548, JQ922546, GU131957, GU131958, GQ868498, KF955416, KF955417, GQ868499, GQ868500, GU131960, GU131961, GU131962, GU131963, GU131964, KF955419, GU131965, KF955421, KF955422, GQ868501, HQ166035, GQ868502, GQ868503, GQ868504, GU131967, GQ868505, GU131968, GU131969, GQ868506, GQ868507, GQ868508, GQ868509, GQ868510, GQ868511, GQ868512, GU131970, KF955427, GU131971, GQ868513, GQ868514, GU131972, GU131973, GQ868517, GU131976, GQ868518, GQ868519, KF955443, GQ868520, GQ868521, GQ868522, GU131977, GQ868523, GQ868524, GU131978, HQ166036, GQ868525, GQ868526, GU131979, GQ868527, GU131980, GU131981, GQ868528, GQ868529, GU131982, GQ868530, GQ868531, GU131983, GQ868532, KF955433, GQ868533, GQ868534, GQ868535, KJ189306, KJ189307, KJ189318, KJ189319, KJ189320, KJ189321, KJ189322, KJ189324, KJ189325, KJ189326, KJ189327, KJ189328, KJ189329, KJ189330, KJ189331, KJ189332, KJ189333, KJ189334, KJ189335, KJ189336, KJ189337, KJ189338, KJ189339, KJ189340, KJ189341, KJ189342, KJ189343, KJ189344, KJ189345, KJ189346, KJ189347, KJ189348, KJ189368, KJ189369, EU482615, EU482616, EU482617, EU482618, EU482619, FJ898433, FJ547088, FJ810419, FJ547068, FJ562104, GQ199867, GQ199857, GQ199858, GQ199859, JF937644, JQ287666, JF937645, FJ850114, JN819402, FJ850113, JF937635, FJ024483, EU596504, FJ024481, GQ199872, FJ410290, FJ024423, FJ024485, FJ547089, FJ024484, EU596503, FJ024479, FJ182002, FJ024482, EU596501, FJ024480, GQ199873, FJ873814, GQ199875, JN819403, KF973453, KF973454, KF973455, KF973456, KF973457, KF973458, KF973459, KF973460, KF973461, KF973462, KF973463, KF973464, KF973465, KF973466, KF973467, KF973468, KF973469, KF973471, KF973472, KF973473, KF973474, KF973475, FJ898448, KJ189351, KJ189352, KJ189353, KJ189354, KJ189355, KJ189356, KJ189357, KJ189358, KJ189359, KJ189360, KJ189361, KJ189362, KJ189363, KJ189364, KJ189365, KJ189366, KJ189367, JN819417, FJ390374, FJ390378, FJ205872, FJ205873, FJ390379, FJ390380, FJ205874, FJ410173, FJ410174, FJ410175, FJ562105, FJ562106, FJ410179, FJ410181, FJ547086, FJ410183, FJ410184, FJ410185, FJ547087, FJ410186, FJ478457, FJ410189, FJ478458, FJ410190, EU482591, EU482592, EU482609, EU482610, EU482611, FJ639735, FJ639740, FJ639741, FJ639743, FJ639794, FJ639796, FJ639797, FJ639802, FJ744701, JN819410, FJ639806, FJ639808, FJ639811, JN819411, FJ639812, FJ639813, JN819412, FJ810415, FJ639814, FJ639815, FJ639818, FJ639819, FJ639820, FJ639821, FJ639823, FJ639824, JN819413, JN819425, KF955407, FJ850100, FJ882579, JN819414, FJ873810, FJ850103, FJ850104, JN819405, GQ199877, GU056029, GU056030, GU056031, GU056032, GU056033, GU131833, GU131834, GU131835, GU131836, GU131837, JN819415, GU131838, GU131839, GU131840, GU131841, GQ868601, EU660396, KC759167, KC692496, KC692497, KC692498, KC692499, KC692500, KC692501, KC692502, KC692503, KC692504, KC692505, KC692506, KC692507, KC692508, KC692509, KC692510, KC692511, KC692512, KC692513, KC692514, KC692515, KC692516, KC692517, KC762649, KF672759, KF672761, KF672762, JF459993, KF672764, EU863650, HQ332177, HQ332181, HQ332180, HQ332183, HQ332179, DQ285561, AF311958, AF311957, EU280167, AY277665, AY277664, AY277659, KC131141, KF289073, KJ189315, KJ189314, KJ189313, KJ189312, HQ166037, HM631853, GU131984, GQ868539, GQ868537, GQ868536, FJ461303, EF122232, EF122231, DQ672564, AF513110, DQ193572, EU081262, M87512, KF955428, KF955420, AY206457, AF514883, AF514885, AF514876, AF514878, AF180818

**HEV VIPR database Selection**

MH992007, MF444058, MF444057, MH377722, MH504140, MH809516, MH504142, AB193178, MF444040, MF444073, MH992008, AB220971, MH992001, KC492825, LC314156, MH504151, JF443726, MH504148, MN646691, MF346772, MH504157, MF444138, MF444030, MH504150, MF444047, MF444117, AB437316, MF444048, MF444077, MH992010, AB080575, HQ389543, KC618403, LC037955, MF444104, MF444064, MF444097, LC022745, JQ679013, MF444096, MH377724, MN401237, AB521805, KU176131, MH504163, MH504132, MF444130, MH377725, AB437317, KY436507, FJ653660, JQ013795, AB193177, MF444035, MF444087, AB074920, MF444109, MF444084, MF444050, MF444108, MF444106, MH504133, LC387631, AP003430, MH504149, MF444088, MF444105, MK089849, AB097812, EU495148, JQ679014, KR872415, MF444072, JQ655734, MF444041, MF444056, MF444082, HM439284, MF444051, MF444054, MF444029, MF444093, MF444055, MF444061, MF444079, MF444037, MF444123, MH504143, MH991996, MF444033, MF444125, MF444067, MF444042, MF444101, MH504127, MH377721, X98292, HQ634346, MF444043, MF444134

**HNoV, genogroup II VIPR database Selection**

KJ196282, KP784694, KJ196287, AB933700, AY502020, AB933677, JN400613, MG049692, KJ196291, AB933676, GQ845024, MF405169, KU561248, KP784691, KU561252, NC_044045, KP784693, KJ196294, KT970369, LN854572, KJ196278, KJ196296, KJ196288, KJ196281, KJ196283, KJ196299, KT970375, KP784692, KT970373, KT380915, KT970372, LN854565, KP784695, KU561251, KJ196295, KJ196293, KT202797, KJ196280, KT202796, KJ649705, KJ196276, LN854570, NC_039475, KP784696, KU561254, KX079488, KP998539, LN854566, KU561249, KT202795, KU870455, KT970377, KU561250, KJ196277, KT202794, LN854569, KJ196285, LN854568, KU561253, NC_044046, KJ196290, LN854567, KU561255, KT970374, AY502023, KU561256, KJ196297, KT202793, KJ196289, KT202798, KT970371, KP784697, KT589391, KJ196284, KP784698, LN854571, KT970376, KT970370, KJ196279, KJ196286, KF429790, AB933648, KY865306, LC145787, MK762560, MG746035, MK775032, KM198573, MK282256, KF429785, MK762633, KF712499, KY947549, GU980585, MH218597, MK754442, KC409272, KC409249, KJ685413, MK775029

**JEV GenBank All**

JN381838, KT957421, JN381872, JN381830, JQ086763, JN381834, AF075723, HQ652538, AB241119, JN381867, AY184212, AB551990, AB569988, AF217620, KM658163, AB471669, AY303796, AB051292, KY927815, KF297916, HQ223286, M18370, JF706283, JN381853, AF080251, KY927818, AY316157, GQ902063, JF915894, KY078829, JN381863, AB551991, AF045551, KT229572, L48961, HQ223287, JN381843, JF706270, HQ223285, GQ902062, GQ902061, KM677246, KT957420, AY303794, KT229575, JF706279, HE861351, JN381868, JN381870, KY927816, U47032, AF098736, EF623987, JN381873, AB241118, JN381848, JN381866, AF221500, AY303792, GU205163, KF667311, GQ902060

**HPgV-1 GenBank All**

AF121950, D87255, AF081782, D90600, LT009483, LT009487, LT009485, LT009494, AY196904, MH053119, LT009481, LT009478, HGU44402, LT009479, AF309966, MK291245, MK291244, AF031827, MH053115, KP259281, MH053120, HGU45966, MH053118, MH053121, HGU63715, LT009486, AB003289, LT009489, AF104403, JN127373, D90601, AB013501, MH053116, D87711, HGU36380, AB008335, D87263, D87712, D87713, KM670109, D87262, D87709, KM670096, LT009490, AB008342, KM670099, D87714, LT009480, KP710601, KM670110, LT009482, D87710, AB021287, KM670097, LT009484, KP710599, HQ331235, KM670100, HQ331234, LT009488, KM670107, HQ331233, KM670108, KP710604, KM670102, KM670106, KC618400, D87708, AB008336, KM670098, HGU94695, KP710605, KP710600, KC618401, MH746815, KC618399, AB013500, KP710598, KC618398, KM670101, MH053117, KP710602, KP710603, AB003288, KP710606, AB018667, HGU75356, AY949771, D87715, AB003290, AB003293, AF006500, AB003291, AB003292, MH179063, LT009493, MK291243, LT009492, LT009491, AF121950

**MNV GenBank All**

FJ446719, JN975495, EU004682, DQ223042, EU004668, AB435515, EU004680, JF320653, JN975498, EU004675, JF320647, DQ223043, EU004671, FJ446720, EU004674, EU004670, EU004681, EU004658, EU004677, HQ317203, EU004667, JN975493, JF320652, EU854589, JN975496, EF531290, AB601769, JN975494, JQ237823, EU004678, EU004663, DQ911368, EF531291, EU004683, EU004679, EU004664, DQ223041, EU004673, DQ285629, EU004656, EU004659, EU004661, EU004662, AY228235, EU004655, EF014462, EU004654, EU004657, JF320648, JF320649, JF320650, JF320645, JF320651, JF320646, EU004669, EU004665, EU004672, EU004660, AB435514, JF320644, EU004676, JQ658375, EU004666

**Canine KoV GenBank All**

KF924623, MN449341, KC161964, MH052678, JN387133, JN088541, MF062158, JQ911763, MK201776, MH747478, MK201779, MK201777, MK201778, KM068051, KM068048, KM068050, KM068049, KF831027, MF598159, KM091960, MK671314, MK671315, MH159813, MH159814, KJ958930

**FMDV-A GenBank All**

JF749843, AY593766, AY593770, AY593756, AY593793, AY593788, AY593782, AY593758, AY593760, AY593771, AY593752, AY593759, GQ406251, HQ832577, HM854024, HQ832576, HQ832585, KJ754939, HQ832591, HQ832584, HQ832589, AY593761, AY593794, AY593768, AY593775, AY593803, AY593790, AY593789, AY593792, AY593767, AY593776, AY593779, AY593755, JN099699, EF494487, JN006722, KM268896, KJ933864, AY593765, HM854022, JF749848, HQ832586, HQ832592, HQ832580, HQ832587, AY593773, AY593787, AY593786, AY593802, AY593801, AY593785, AY593784, AY593783, AY593769, AY593753, AY593757, AY593781, AY593780, AY593778, AY593754, APHA12CDR, AY593777, AY593774, AY593751, JN099698, JN099697, JN099688, JN099695, JN099694, EF494488, EF117837, JF749841, EF494486, KC588943, GQ406250, GQ406249, GQ406252, GQ406248, GQ406247, HQ268509, HQ632773, HM854023, AY593764, AY593763, AY593762, X74812, AY593772, AY593791, HQ832590, HQ832583, HQ832579, HQ832578, HQ832581, HQ832582, HM854025, HM854021, HQ832588,

**FMDV-O GenBank All**

AY593824, JN998086, FJ461345, DQ248888, AY686687, EF552691, JX066664, JX040492, KF694737, AY593813, AY593822, KJ206910, KJ825804, AY593826, KF112882, HM191257, GU384683, HM008917, EU448369, AY593817, GU125647, AY593833, KF112880, KF112885, JX869177, HQ632769, AY593827, AF511039, AY333431, DQ478937, KF112888, FJ461344, AY593812, HQ412603, HQ113232, KJ206908, HQ632768, AY359854, FJ175664, KF112879, JQ900581, AY593830, AY317098, AY312588S2, GU125650, KF501486, HQ009509, AY593834, AF377945, DQ119643, KF112886, EU140964, HQ632770, KF501488, KM268895, HQ632771, JX869178, AY593825, JF749851, HQ632772, HQ268524, AY593823, AY593821, KF985189, KJ206909, JX040501, AY593828, AY593829, KJ825801, AB079061, AY593814, JX040488, AY312586S2, DQ404179, JX040497, EF552696, JX040493, JN998085, KR265072, JX040490, FJ542366, DQ404169, DQ404170, AJ320488, FJ542370, EU214601, JX040491, DQ404164, HM055510, KR265074, JX570643, EF614457, DQ404166, EF552695, DQ404162, JX869186, JX570653, JX570652, DQ404168, AY593820, KF694740, DQ404165, EF552690, EF552694, GU125648, KJ825808, AY593815, JX869179, KF694731, JX869182, AY593818, JX570644, JX869181, EU448381, JX570654, EU448379, JX869185, DQ404175, DQ404167, AY593811, JX869180, JX570655, AF154271, DQ404159, EU448376, FJ175662, JX040495, EF552692, AJ633821, EU448377, AJ539140, JX869184, EF552697, JX570647, KR265075, KM257062, GU384682, FJ542369, KM257063, JX570641, KF694733, FJ175665, KJ825806, AJ539137, KJ825807, JX040487, AJ539139, EF552689, KM257065, KF112887, JX947858, DQ478936, EU448373, KF112881, EU448372, DQ404174, DQ404160, JX570645, DQ404176, JX570640, JX869187, EU448368, KC503937, AY593816, KJ825803, KF694743, AY593836, EU448378, JX570638, EU448375, JX040499, JX869188, KJ825805, KF112883, FJ542365, KF694745, KM257064, JX570648, JX570650, EU448371, AF506822, FJ542372, KF694739, KF112884, FJ175661, EF175732, EU448380, JX040485, DQ404173, FJ175663, JX040489, X00871, DQ404161, EU448370, FJ542371, DQ404172, KF694735, JX040498, EF552688, JX040494, EF552693, FJ542368, JX570651, DQ404158, DQ404177, AY593835, JX040486, KF694741, KJ825809, KR401161, AF308157, JX570649, FJ175666, JQ973889, DQ404180, JX869183, JX040496, KF112889, KF694744, KF694736, EU448374, KJ825802, JX570639, KR265073, AY593831, AY593837, DQ404163, AJ539136, GU125649, JX066665, AY593819, AY593832, KF694742, KM257061, KF694732, KF694738, KF501487, DQ404178, DQ404171, AJ539138, AJ539141, JX570642, JX040500, JX570646, FJ542367, HM229661

**RUBV VIPR database All**

AY258323, AY258322, AB928203, AB928205, AB928204, KX291007, FJ211588, FJ211587, CS406445, AB588192, AB047330, AB047329, AB222608, KT962867, KT962871, JN635284, JN635287, KT962864, KT962869, KT962870, JN635288, JN635281, AB222609, JN635283, AB860305, KT962866, KF201674, JN635291, KT962863, JN635285, JN635282, JN635286, L78917, JF727654, JF727653, AB588190, AB588191, AB588189, DQ388281, NC_001545, M15240, DQ085339, AX009468, AF188704, DQ085340, DQ388279, DQ085341, DQ085343, MF496142, AB588188, KT962868, JQ624625, JN635293, JN635294, JN635292, KT962865, JN635295, JQ624624, JN635296, KT962862, AB588193, DQ085338, DQ085342, JN635290, JN635289, DQ388280, X72393, AF435865, AF435866, KU958641, KT000088, KT000089, AF533117

**IAV segments 1-3 GenBank Mammalian selectiom**

JN582058, HQ533878, DQ508870, DQ508902, GQ457545, HQ291989, DQ360837, FJ912981, HQ166041, HQ291980, GQ902830, DQ508838, GU183796, FJ445033, FJ913003, FJ445079, GU271955, JN582066, DQ415292, JQ714243, JQ290173, HM172451, GU907121, DQ415287, FJ912943, DQ415293, FJ912925, HQ291994, HQ291985, FJ912961, AY627898, GQ457544, HQ291988, GU271971, HQ291981, AF348170, GU271963, DQ889682, HQ291984, HQ291987, DQ508886, DQ487334, DQ508926, FJ913009, HQ291977, DQ415286, HM172413, EU263988, EU268224, DQ835310, DQ415288, JQ070789, GQ902822, AX399724, HQ291976, AY626149, M73524, HM114542, AB212051, AY342413, DQ009923, HQ291971, AJ404630, FJ912997, GU183804, GQ983545, GU271987, FJ912937, FJ445071, EU399758, HQ291975, AB462292, JN638726, JQ290185, JQ070777, DQ508878, FJ912949, FJ912913, HM114582, FJ912967, FJ912989, GQ902798, JN588923, DQ508894, FJ445065, HQ291970, HM172438, GQ902790, GQ457546, EU268216, HQ291982, HM006756, HM114598, GU271979, GQ902814, HM114606, V00603, HM114558, HM114550, HQ291991, HM114566, HQ291992, HQ664922, DQ469955, GQ850592, AY627892, GU271947, EF587274, FJ445082, FJ912931, HQ291973, HQ166049, HQ291983, HQ291974, HQ291978, FJ445038, DQ415289, HQ664929, EU434693, HQ291990, DQ508854, FJ912973, DQ508862, DQ415283, AY818126, HQ291972, HM114614, EF467818, DQ415290, AF389115, JQ290169, GU183812, EU597800, JQ070797, FJ445067, FJ912907, DQ415284, FJ445036, GQ902806, FJ912919, EU434701, HQ291986, HQ291993, AY646085, DQ415285, DQ415291, HQ664917, HQ291979, AB434397, JQ350549, GU086014, JF411835, FJ789824, JN375001, AY790311, GQ229329, EU516317, JN809099, GU135896, JQ738184, GU135946, JN409420, EU015993, AY747616, JN375007, JN375019, JN375025, JF820274, AB620196, GQ422427, EU273802, JN375028, JN375022, FJ200425, AY700215, JQ914802, GQ422428, GQ229337, JN409396, JQ689137, JQ738168, HM440066, JQ689145, EU273805, HM215166, HM440098, EU924268, JN375009, GU086070, JN809093, JN375282, JQ689113, JF411836, JN375000, JN809102, GQ229377, JN375286, HM440138, JQ277166, EU053138, HM440074, GQ484358, JF411837, JQ220533, JQ689089, HM210867, DQ469971, GQ161153, JQ914792, FJ536816, JN409404, JN809100, JN809108, HM440106, AB441177, JQ738160, JQ220534, EU086333, GQ229321, GQ229305, JQ914782, DQ139325, AB620188, AY619970, AB434285, HM189301, GU135915, HM215158, EU116038, DQ150422, JF713871, AB434293, AB598483, FJ200417, AB598481, JN375002, JQ738152, JQ365121, JF411842, JN656959, AB620172, AB434381, JF411838, GU135954, JN940419, JN375017, EF551050, AB600245, JQ624664, AB434309, DQ280205, GU135938, GU086022, DQ150430, HQ825240, JN809094, JQ689129, GQ422382, EU502892, DQ280213, JN809110, JN375006, GU135970, JN656964, GU086134, JF316640, AB434413, GU086062, EU053130, JF714033, JQ350543, HQ853340, JF820282, AB434389, JN409428, FJ410137, JQ689121, EF612749, JN809109, EU502906, AB434349, HQ825187, JN409436, DQ280221, GU086038, JN375026, DQ280245, GU135962, EU604691, HM440154, EU502898, JQ365116, GQ161116, JN375285, JN409412, GU135907, AB434405, AB434317, JN809098, JQ738144, JN809107, JN375284, JQ350527, GQ161101, AB598484, FJ611896, AB598485, JN375004, JN809103, AB434373, EU273803, DQ997252, JQ350535, JN375024, EU258950, DQ469963, HM223601, JQ350555, GU086046, JF714017, AB434341, GQ422426, AB434357, GU086054, JN375029, GQ422430, EU273804, AF251434, JN375016, EU004447, GU086006, AB434333, JN375005, HM440146, JN375283, AB600246, JQ914807, DQ469979, AY619954, AB598482, HQ840335, GQ422429, JQ914787, JQ738176, AB434301, EU516309, AY619962, AB600243, FJ789830, HM125979, EU086332, FJ157988, AB600244, JN809096, DQ280229, JN375020, JQ350519, JF411839, DQ280237, HM210859, JQ914817, JF411841, DQ280189, HM440130, GQ422431, DQ469987, JN809106, GU135930, DQ280197, HM440122, HM440090, JN809104, HQ541647, JN375023, AB600242, JQ914797, FJ830852, JN375027, HM440114, AB598480, HQ825179, HM440058, JN375008, GU135923, HQ825232, JN809095, AB434325, EU086316, JF290388, HQ541671, EU826550, JQ914812, HQ825195, AY747624, GU086078, JN375021, JN375003, JF411840, JN809097, JN409388, JN809101, EU258942, HM440082, HM215174, FJ374512, AB620160, JN809105, GU086030, HM223593, JQ220532, JN375018, EU794524, EU794532, AB591847, EU794564, EU794516, FJ375221, EU794492, EU794540, EU794572, DQ222920, EU794508, JQ228396, EU794556, EU794548, GU571147, AY855338, JN247616, GU433376, GU433360, GU433368, JN247576, JN247592, GU433351, JN247608, JN247600, FJ560890, JN247584, EU420053, DQ643980, EF178522, EU420045, AM744956, HQ454985, EU532420, EU826558, GQ176137, FJ912938, DQ508839, FJ912944, GQ902799, HQ166042, HQ291951, DQ508871, HM114551, HM114543, DQ415302, EU268217, HQ200601, HM172394, AF348172, HQ291959, FJ912920, FJ912962, DQ889683, JN638731, FJ912926, JQ070790, DQ415297, DQ508887, HQ291950, FJ912974, JQ070782, GQ457567, AX399725, HQ291946, HQ664936, FJ912932, FJ913004, GQ902815, DQ508895, HQ291964, DQ415298, HM006757, HQ291958, HM114599, FJ445055, GU271964, HQ533876, HM114615, FJ912950, AF389116, GU271980, GU271988, GU183805, GQ902807, EU597801, GQ850593, HQ291954, DQ508903, FJ445026, JN582057, EU263987, HQ291966, FJ913010, AJ404634, HQ291963, FJ912908, GQ457566, DQ415301, DQ508863, FJ445084, GU271956, DQ415299, AY627897, FJ445037, HM114559, EF467819, AY627891, FJ445068, AB212052, DQ835311, EU399757, GQ902791, JN582065, HQ291969, DQ415303, DQ508855, GQ902823, AY340083, HQ291968, DQ360838, HM172359, DQ508879, FJ912998, HQ664937, HQ291945, HQ291952, DQ469956, HQ291953, JQ070798, HQ291965, JQ290170, HQ291948, HQ291949, HQ291967, FJ912968, AY626148, GU183813, HM172369, DQ415295, HQ291962, DQ415296, HM114607, DQ009922, AY818129, GU271948, HQ166050, AY646084, FJ912990, HQ291947, GU907118, AB462293, FJ445043, GQ457565, JQ290174, EU434700, FJ912914, HQ291961, GU271972, JQ290186, FJ445034, DQ487333, EF587275, DQ415300, HQ291956, DQ508927, J02151, JQ714244, GU183797, HQ291957, HQ664919, HM114583, EU268225, GQ902831, GQ983546, HM114567, FJ445083, EU434692, FJ912982, DQ415304, DQ415294, M25935, HQ291960, HQ291955, DQ469988, FJ410138, GU086071, GQ161154, HQ825180, JN375056, JN375046, JQ914808, EU015992, GU086015, JN656960, AY619955, GU086135, HM440089, GQ422424, GU086023, GU086031, GU135947, HQ825233, JN375053, AB598488, EF612748, JQ220537, AB600564, HM440081, DQ280238, AB434302, GU135963, GU135955, HQ825188, GU135916, AB620197, JQ220536, EU258949, AB600563, HM440113, JN375047, AB434286, AB600561, EU273799, AB434406, DQ280230, JN409421, JN375044, GQ161102, JN809111, JN809124, JN809122, GU086079, JQ350528, EU273798, HM440129, JQ689130, JN809125, JN375055, JQ738145, JN375054, HM210866, DQ150431, EU924269, JQ689090, AB620173, EU692892, AB434382, EF551051, GQ422383, HM215165, JQ350550, GQ229316, HM440121, JQ689122, AB600560, GQ229372, EU502907, DQ280246, AB434374, JQ914813, JQ350556, JN375043, FJ789831, JN809121, JN375287, HM440057, HM440065, HQ315645, JN375065, GU135897, GU086055, JN375050, FJ789825, DQ469972, AB434342, JN809126, JN375289, GU086063, AB620189, JN375060, GQ229324, FJ200426, JN409429, HQ541670, JN375288, HQ315644, AB598487, EU692893, JN375052, JQ365117, HM440097, GU086047, GQ422423, JF820275, GU086039, EU273801, GQ422420, EU604692, HM440105, HM210858, EU004446, JN940420, AB434334, FJ200418, JQ624665, JN809114, JN375062, GQ422421, JQ350520, JN409413, FJ830853, GQ484357, HQ825196, GQ422425, AB598491, JN375042, FJ536817, JQ738169, JN375063, GU135908, AB598489, AB441176, AB598490, AB434358, JN809127, EU516318, EU516310, HM215173, HM223592, HM125980, DQ139327, JN809115, JQ277165, AY700214, HM440073, JN809112, JQ738153, DQ280222, EU826549, JQ914793, HM440145, GU135971, AB434310, JN809118, AB434350, JN809117, AY747623, JN656965, DQ280198, EU692896, JQ738185, HQ541646, JQ350536, AB434318, JQ365122, JN809120, JQ914818, JN809123, EU502899, AB598486, JN809116, GU135939, EU692890, JQ914783, JQ914798, AB434294, GU135931, JQ914788, HM223600, EU273800, JF714018, JN375045, EU086330, JQ220535, GU135924, AB600562, JN809113, JN375290, AB620161, JQ689138, AY619963, HQ840336, JQ738177, JQ350544, AF251429, AB434414, JN375051, JF713872, JN409389, JQ738161, GQ422422, JN375057, JN409397, EU258941, GQ229300, EU692891, EU502891, DQ997251, HM440153, GU086007, HQ825241, DQ150423, AB434390, AB434326, HM215157, EU086314, HQ853341, EU053139, JN375048, EU086331, EU116037, JQ914803, JF820283, JQ689146, HM189302, JQ689114, AB434398, AY619971, DQ280206, AY790312, GQ229332, JN809128, JF316641, JN375064, FJ157989, AY747615, JN375061, DQ469980, FJ611895, DQ469964, JN375049, JF714034, JN409437, JN375058, GQ161117, JN375059, JF290389, EU053131, DQ280214, JN409405, JN809119, FJ374513, EU692894, HM440137, JN375291, JQ228395, GU571148, EU794557, EU794533, EU794549, EU794573, EU794493, AY855339, EU794541, EU794525, EU794517, DQ222919, FJ375233, AB591846, EU794565, EU794509, JN247593, GU433359, JN247609, JN247617, GU433350, FJ560889, JN247585, GU433367, GU433375, JN247577, JN247601, EF178523, AM744955, GQ176138, DQ643979, EU826557, EU420052, EU532421, EU420044, HQ454984, FJ912975, AX399726, GU271957, HQ292026, HQ292025, EU268226, DQ508880, HQ664918, DQ415314, AJ404637, HQ664923, FJ912909, GU271989, HM172342, HM114584, DQ508928, GQ850594, FJ912999, FJ445032, M26079, DQ009921, V01106, DQ415315, HQ664930, FJ912991, FJ912927, EU263986, HQ292032, FJ445056, HM114552, FJ912951, HQ292036, HQ292038, HQ292022, HM114600, DQ415305, FJ912933, DQ415308, DQ415307, JQ290184, HM114608, GQ902800, HM006758, GQ902792, HM114568, JN582064, DQ508864, HQ292027, HM114544, FJ912945, GU271973, AY818132, HM114616, FJ912983, JN638730, EF467820, AY342418, FJ913005, GQ902808, HQ533875, DQ508896, JQ290168, AY627890, AB212053, GQ902816, FJ445072, HQ292043, FJ912939, JN588893, HQ292031, AY646083, HQ292029, JN582056, GQ902824, HQ292033, EU434699, EU268218, AY626147, HQ292042, DQ508872, FJ912921, GU271981, GQ902832, HQ292044, EF587276, HQ292023, HQ292021, GQ457564, AF389117, DQ835312, DQ415311, JQ290178, FJ445078, DQ415313, HM172310, GU271965, JQ714245, FJ445063, HM114560, HQ292035, JQ070781, EU399756, DQ415310, DQ508840, HM172315, DQ415309, JQ070796, GU183814, HQ292040, HQ166051, FJ445076, JQ070788, GQ457563, HQ292024, FJ913011, DQ415306, DQ487335, DQ508904, DQ508888, DQ360839, GQ983547, DQ415312, GU907117, EU434691, GU183798, AY627896, HQ292037, EU597802, GU183806, GQ457562, GU271949, HQ292041, DQ508856, HQ292028, HQ292020, HQ292039, AB462294, FJ445069, FJ445035, DQ889684, HQ292034, HQ292030, AF348174, HQ166043, FJ912969, FJ912915, DQ469957, FJ912963, GQ422384, DQ150432, JN809146, FJ410139, JQ689131, EU116039, JQ365123, JN375081, HM215156, JQ350551, AB434351, JQ350521, JN940421, GU135964, GU086024, JQ220538, HM440128, AB600846, DQ469981, JN375096, HQ825181, HM440064, EF612747, HQ541645, JQ914814, JQ689115, JF714035, DQ280223, JN375076, JN409414, HQ825234, GU086040, AB620190, GQ422415, JN809134, GU086064, DQ280215, AB598492, GU135956, GU135909, AB620162, AB434295, EU086313, HM189303, HM440144, AB434399, JN375095, JQ277164, AB434343, JN809135, JN409422, FJ157990, FJ536815, HM440112, JN809143, EU086329, AY700213, JQ914809, JN375098, DQ469965, JQ738162, GQ484359, EU273795, JN375074, JF346153, HQ825242, AB600847, GQ161118, GU086072, AB598497, JQ624666, FJ200427, JN375090, DQ280247, JN409438, JN809142, GU086016, EF551052, HM440096, JQ914784, HM223599, JN809144, DQ997257, HM125981, JN809129, GU086080, JN809136, JF316642, JN375092, JN375294, AB434415, AB434287, GQ161155, AB600848, JN809133, GQ161103, JN809140, JF346154, HM440152, AY790310, JN375099, HM215164, JQ738186, JN809137, AB598495, JN375075, AY619956, JN375097, JQ220539, JN409406, HM440056, DQ280231, AB434383, AY619964, GU086048, AB434391, JQ738146, JQ689123, GQ229376, JF346158, JN409430, JF290390, EU516319, HQ541669, JN656966, HM440072, DQ280239, DQ150424, AB434311, HM210857, JQ914799, FJ830854, DQ280199, EU502908, GU086032, JN375088, AF251433, HQ825197, HQ840334, AB441175, JF820284, JQ738178, JN375077, JF346152, JF346155, JN809141, HM215172, JN375295, AY747614, HM210865, JQ350537, GQ229320, HM440104, JN375073, GU135932, EU015991, GU086008, DQ469973, EU004445, DQ139326, JN375101, GU135948, JN375093, JN375080, GU135925, GQ422418, AY619972, JN375293, JN809139, JN375296, JN809138, GU135898, JN809132, JQ914819, JF714019, AB434407, HM223591, GQ422414, HQ853342, EU826548, HM440120, JN375292, JN409398, DQ280191, AB620198, JQ350529, GU135917, FJ789826, GQ229304, AB598493, EU502890, JN809131, JN375091, GQ229328, AB434319, JN809130, JN375072, EU086328, JQ365118, AB434327, DQ280207, JQ738170, GU135940, JN375100, HM440136, EU924270, AB600844, AY747622, JQ914789, JN375079, JN375089, AB600845, FJ374517, AB598494, EU604693, EU273797, JQ914794, JN809145, HQ825189, JQ738154, AB598496, JQ689139, GU086136, JQ689091, GQ422417, DQ469989, JQ914804, EU258940, JF346151, AB434303, HM440080, JN409390, JQ350545, AB434375, EU516311, JF713873, AB434359, FJ611897, JQ689147, JQ350557, JF820276, GQ422419, JF346156, EU273794, JN656961, AB434335, JQ220540, JF346150, GQ422416, EU258948, EU502900, EU273796, AB620174, EU053132, GU086056, HM440088, JN375094, GU135972, JN375078, GQ229336, EU053140, FJ789838, FJ200419, EU794558, EU794574, AY855340, EU794534, EU794494, EU794566, EU794550, EU794510, DQ222918, EU794526, JQ228397, EU794518, AB591845, EU794542, GU571149, FJ375228, JN247618, GU433349, JN247602, JN247594, GU433374, FJ560888, JN247586, GU433365, JN247578, GU433358, JN247610, DQ643981, GQ176139, HQ454983, EF178524, AM744954, EU420051, EU532422, EU826556, EU420043.

**RABV VIPR Database All**

LT909550, MK540971, KX954123, MN233902, MG458313, KU198464, KU198461, JQ944707, MK540895, KU198465, MK540891, LM645044, LM645041, LM645056, LM645052, LM645054, MN233945, KU198471, KU198462, KU198463, MN234045, KU198478, MN233970, MN233969, MN234053, MN234005, MK540924, MK540936, MK540957, MK540947, MK540984, MK540985, MK540978, MN234009, MN233941, MN234055, KU198473, KU198477, MN233933, MN233999, MN234010, MN234008, MN234033, MN233994, MN234001, MN234003, MN234041, MN234015, MN233942, MN234039, MN234044, MN233998, MN233973, MN234046, MN234035, MK540927, MK540898, MK540905, MK540901, MK540912, MK540967, MK540992, MK540965, MK540921, MK540897, MK540923, MK540929, MK540964, MK540913, MK540914, MK540920, MK540903, MK540893, MK540889, MK540902, MK540900, MK540934, MK540977, MK540904, MK540915, MK540911, MK540919, MK540896, MK540950, MK540926, MK540966, MK540951, MK540916, MN233914, MN234018, MN234019, MN233974, MN234027, MN233919, MN233917, MN233977, KU198466, MN233962, MN233898, MN233949, MN233947, MN233959, MN233958, MN233956, MN234013, MN233899, MN233989, MN234023, MN233984, MN233987, MN233988, MN233948, MN233975, MN234016, MN233900, MN233985, MN234028, MN233922, MN233978, MN233976, MN233963, MN234017, KX036366, KX036361, LT598537, LM645017, LM645020, MN233954, KU198476, LT598540, MK540907, MK540887, MK540899, LM645036, LM645032, LT598543, LM645028, LM645035, LM645031, KX036363, KX036364, LM645026, LM645018, MN233910, MN233952, MN234022, MN233921, LM645016, MK540917, KX148105, MK541005, MK540975, MK540993, MK540976, MK540995, MK540989, MN233903, MK540990, MK540940, MK540925, MK540939, KU198468, MK540918, MK540982, MK540987, MK540948, MK540955, MK540969, JQ685938, JQ685968, LT909549, KX148230, KX148231, KX148229, MF630920, LR812034, LT993243, EU182347, LR812028, LR812027, AB781935, CS614408, EF206717, JX276550, NC_001542, JQ685894, LT909544, KX148111, KX148102, HQ450386, KX148112, AB085828, GU565703, GU565704, JQ685899, DQ099524, HQ891318, DQ875050, JQ944709, LT839616, HM535790, DQ099525, JQ685943, MF197745, KT006769, KX708503, KX708502, KX708499, KX708504, KX708501, KX708500, JQ685929, JQ685975, JQ685954, KM594026, KM594027, KM594028, KX148101, KF154998, MG458316, MG458317, MK760770, MK760769, MK760768, AB009663, LT909530, KX148214, KX148216, AB517659, KC737850, LT909548, KX148218, KX148219, KX148212, KY860606, MK760766, JQ944708, KY860605, KX148107, KX148232, KX148237, KX148236, KX148233, KX148234, KX148235, KX148191, KX148192, KX148142, KX148160, MK598339, MK598345, MG458311, KX148144, KX148141, KX148143, MK598398, KX148140, KX148138, KX148139, JQ944705, MF197741, MG458312, KX148136, KC595282, KC595281, KX148181, KY860599, LT909526, KX148186, KX148185, KY860611, KY860612, KY860609, KX148164, KX148167, KX148184, KX148170, KX148171, KX148173, KX148190, KX148189, KX148188, LT909540, KX148183, KY860595, KY860603, KY860607, KY860592, KY860590, KY860593, KY860588, KY860586, KY860589, KY860583, KY860596, KY860594, KY860597, KY860600, KY860584, KY860591, KX148176, KX148137, KX148145, LN879480, KX148104, KY765901, KX148159, MG458307, MK760674, MK760670, MK760751, MK760669, MK760731, MK760753, MK760707, MK760723, MK760712, MK760693, MK760732, MK760762, MK760703, MK760749, MK760721, MK760745, MK760748, MK760716, MK760726, MK760720, MK760758, MK760738, MK760699, MK760671, MK760704, MK760683, MK760695, MK760742, MK760698, MK760715, MK760677, MK760701, MK760673, MK760710, MK760691, MK760687, MK760680, MK760708, MK760740, MK760724, MK760694, MK760759, MK760743, MK760668, MK760764, MK760739, MK760688, MK760728, MK760686, MK760692, MK760678, MK760672, MK760667, MK760725, MK760752, MK760685, MK760765, MK760733, KP997032, KT728348, KT728349, KC595283, KC595280, MK760700, MK760706, MK760763, MK760741, MK760722, MK760705, MK760689, MK760750, KX148161, KX148238, KX148244, JQ685970, MK760734, MK760681, MK760697, MK760709, KF620488, KF620487, KF620489, JQ944706, KF155000, KY860610, MK760757, MK760676, MK760682, MK760679, MK760675, MK760690, MK598343, AB699220, MK760719, GU345747, KX148267, GU345746, KF726852, KY649620, KC252633, MH267792, KX148172, KM016899, FJ712196, GU647092, JQ946087, AB569299, MG458320, KY775603, AY956319, EF437215, KF154996, LT909541, LT909539, HE802675, HE802676, KY775604, MG099711, LC571946, KU946961, KJ466147, EF564174, MN857169, KX148262, KX148261, KX148263, JQ970480, KY451767, EU643590, MG458319, KT336434, KY860602, KX148211, MT454644, KR906751, KR906747, KR906757, KX148210, KX148209, MN857167, MN857168, MN857171, MN857170, MK760744, KM594029, MG458305, EU293116, KM594037, KM594038, JQ685944, JX088694, KR906748, AB645847, KX148213, JQ685967, KF154999, MH671332, KX148108, KX148221, KX148220, MG458308, KX148222, MK920923, JQ685955, AB608731, JQ685895, KX148180, KX148177, KX148179, KX148249, KX148250, KX148257, KX148255, MG201920, AB981664, KX148248, AB981663, JQ730682, EU293121, KX148254, KX148253, KX148251, KX148252, MN075931, KX148247, JN786877, KM594032, MG201921, MG201919, GU358653, AB519642, EU293113, MG458314, KX148109, KM594043, KM594041, KX148100, KU523255, JQ685936, JQ685953, KT336437, AB519641, KM594042, KM594040, JQ685905, JQ685963, MF143230, MF143366, MK540768, MK540762, MF143203, MK540698, MK540744, MF143330, MK540758, MK540697, MK540720, MK540734, MK540797, MN418145, MK540750, MN418163, MK540776, MK540738, MK540737, MN418180, MF143206, MF143212, MF143201, MF143218, KY026414, MF143196, MF143219, MF143214, MF143194, MF143193, MF143191, MF143192, MF143210, MF143204, MF143215, MF143242, MF143217, MF143207, MF143198, MF143213, MN418156, MF143315, MF143197, MN418146, MN418177, MK540709, MF143320, MF143332, MF143296, MF143335, MF143326, MK540675, MF143278, MK540775, MF143284, MF143319, MK540719, MK540726, MF143317, MG562519, MG562589, MK540693, MG562562, MG562602, MG562597, MN418150, MF143200, MF143220, MG562596, MG562580, MN418173, MG562594, MG562610, MG562518, MF143337, MF143306, MF143282, MF143341, MK540765, MK540783, MK540791, MN418143, MG562603, MG562552, MG562595, MG562605, MG562565, MF143400, MN418175, MN418165, MK540824, MK540802, MK540858, MK540879, MK540827, MK540855, MK540868, MK540806, MK540853, MK540872, MK540845, MG562532, MK540882, MK540838, MN418153, MN418172, MN418171, MN418181, MK540703, MK540741, MG562573, MK540716, MF143279, MK540733, MK540785, MK540788, MN418155, MN418144, MK540739, MK540696, MK540760, MG562590, MK540694, KY026482, MG562575, MK540761, MK540702, MG562544, MG562599, MG562607, MG562538, MG562547, MG562546, KY026480, MG562560, MG562588, MF143340, MF143338, MN418147, MN418162, MN418160, MN418161, MN418182, MN418158, MN418167, MN418174, MG562521, MK540710, MK540789, MK540790, MK540748, MG562545, MK540729, MG562527, MK540766, MK540728, MK540700, MK540692, MK540707, MK540724, MK540708, MG562525, MK540759, MK540772, MG562530, MK540695, MK540731, MK540691, MG562523, MK540665, MK540682, MG562526, MK540884, MK540688, MG562535, MK540718, MK540712, MK540769, MK540706, MK540713, MG562579, MG562529, KY026481, MK540704, MK540755, MF143286, MF143334, MF143401, MF143397, MF143392, MF143414, MF143291, MF143393, MK540714, MK540740, MF143411, MF143405, MK540792, MK540850, MG562533, MK540721, MG562539, MF143321, MF143323, MK540784, MK540684, MK540796, MF143290, MF143297, MF143293, MF143311, MF143302, MF143313, MF143322, MF143295, KY026427, KY026416, KY026417, MK540685, MK540664, MK540730, MK540727, MK540770, MG562522, MK540689, MK540756, MK540674, MK540779, MF143258, MF143324, MF143301, MF143308, MF143307, MF143318, MF143294, MK540782, MK540705, MG562551, MK540781, MN418142, MF143329, MF143312, MF143316, MG562571, MK540732, MN418154, MF143280, MF143300, MK540798, MK540793, MK540773, MK540735, MN418178, MK540736, MK540763, MK540725, MK540795, MK540711, MG562587, KY026478, MK540752, MN418168, MG562563, MG562576, MG562606, MG562543, MK540764, MF143205, MK540778, MK540799, KY026420, MK540679, MF143385, MF143328, MG562549, JQ685957, MN418179, MF143208, MF143202, MG562550, MK540749, MF143333, MF143299, MF143304, MF143327, MN418148, MN418170, MK540751, MN418176, MF143209, JQ685901, MK540787, MN862283, JQ685952, JQ685918, JQ685965, MN418149, JQ685921, KY026421, MF143277, MK540794, KM594031, KM594030, JQ685909, KM594033, KM594035, KM594036, KM594034, JQ685925, JQ685917, MG458304, JQ685911, JQ685932, JQ685906, JQ685926, JQ685941, JQ685964, JQ685946, JQ685960, JQ685933, JQ685913, JQ685974, JQ685945, JQ685961, JQ685950, JQ685934, JQ685898, JQ685907, JQ685951, JQ685931, JQ685973, JQ685919, JQ685915, JQ685910, AY705373, JQ685902, JQ685922, JQ685900, KM594023, JQ685942, KM594025, JQ685947, KX148118, KX148217, KF977826, KX148241, KX148240, KX148243, MK598361, MK598374, MK598395, MK598381, MK598378, EU293115, KX148128, KX148149, KX148148, LT909537, KF154997, KX148154, KX148146, KX148152, KX148153, MF197742, MG458310, KX148131, KX148129, KX148120, KX148121, MG458306, KX148115, KX148116, KX148117, KX148114, KX148113, LT909547, KX148134, KX148123, KX148135, KX148151, KX148187, LN879481, KX148150, KX148125, KX148132, JQ944704, MF197743, MK598349, KX148158, KX148157, MK598346, KX148155, KX148156, AB635373, KX148193, LT909546, DQ875051, JN234411, GQ412744, AB128149, LT909531, KP723638, KX148200, LT909551, KX148202, KX148198, KF155001, KX148196, KX148195, KX148194, LT909529, KX148197, KC196743, KX148242, AB517660, KX148266, MF197744, KX148246, KR906741, KC171643, KC171644, MK760747, MK760684, MK760760, KY860613, KC171645, LC553558, KY952220, KY964322, KM272192, KY982923, KY175230, KY175229, KY964323, KY210257, JX473840, JX473841, JX473838, LT909536, MT454645, MT454651, MT454649, MT454650, MT454646, MT454647, MG458318, LT909534, KR906779, KR906745, KY210295, KY210308, KY210268, KY210271, KY210276, KY210262, KR906786, KR906754, KY210225, KR906772, KY210222, KY210273, LC029889, KY210259, KR906788, KR906767, KR906756, KF155002, KY210260, KR906775, KR906750, KY210275, KY210228, KR906783, KY210221, KY210287, KR906740, KX148205, KY210263, KY210245, KX148207, KR906791, KR906759, KY210279, KR906769, KY210282, KY210253, KR906777, KY210264, KR534219, KY210285, KY210223, KR906762, KX148206, KR906766, LT909528, KR906768, KX148103, KX148204, KT336433, MT454633, MT454642, MT454632, MT454640, MT454638, KR906739, KX148208, JQ685916, MK760702, MK760735, MK760729, KX148228, KT336435, KM594024, KR906749, KR906736, KR906764, KR906735, KT336436, KX148203, MT454648, MT454631, MT454634, KT336432, MT454637, KX148201, KY952219, FJ866836, KC169986, JN609295, FJ712193, FJ712194, MK689676, KC193267, KJ564280, EU549783, KJ004416, JQ970483, KY780299, JQ970481, JQ970486, JQ970482, MN175989, GU345748, KX148265, KR230090, JQ423952, MK577649, KR230089, FJ866835, JQ970487, KC977995, MN186249, MN186250, JQ970484, MK689674, KF726853, MK689675, MG458321, HQ450385, MG201922, MG201923, JQ647510, KC762941, KX148227, LT909545, KX148226, KX148225, MK760761, JQ970485, EU293111, MN234031, MN233982, KX148264, KY026469, KY026460, KY026476, MK540717, MG562537, KY026418, KY026447, KY026448, KY026441, JQ685903, KM198893, EF542830, KM594039, AB362483, FJ712195, KX148245, JQ685920, JQ685971, KX148260, KX148259, KY997452, KU198460, LM645047, LM645048, LM645040, LM645050, LM645046, LM645055, LM645053, LM645037, LM645045, LM645049, LM645051, LM645043, LM645039, LM645042, MN233946, MN233901, MN233971, MN233968, MN233992, MN234051, MN234000, MN233995, MN233996, MN233972, MN234054, MK540938, MK540922, MK540931, MK540963, MK540959, MK540932, MK540935, MK540981, MK540983, MK540988, MK540943, MK540953, MK540979, MN234011, MN234012, MN233940, MN233944, MN418166, MN234034, MN234006, MN234047, MN233932, MN233924, MN233926, KU198474, MN233930, MN234048, MN234042, MN233904, MN233905, MN233935, MN234050, MN233936, MN234052, MN233937, MN233938, MN233939, MN233943, MN233993, MN234049, MN234004, KU198472, KU198475, MN233934, MN233928, MN233929, MN233931, MN233990, MN233925, MN234002, MN233997, MN234040, MN234038, MN234007, MN233991, MN234037, MN234036, LT598539, LT598541, LT598538, MN234043, MN233927, KU198479, MK540894, KX148224, MK540986, MK540930, MK540890, MK540892, MK540933, KX148106, MK540944, MK540946, MK540954, MK540956, MK540928, MK540968, KU198469, MK540972, MN234020, MK541012, MN233913, MN233915, MN233916, MN233918, MN233950, MN233953, MN233965, MN233957, MN233966, MN233964, MN233986, MN233980, MN233979, MN233967, MN233951, KU198467, MN233981, MN233983, MN234014, MN233960, MN233961, MN234021, MN234030, MN234025, MN234024, MN234026, MN234029, LM645027, LM645015, KU198470, LT598542, MK540888, LM645034, LM645019, LM645023, LM645021, LM645038, LM645033, LM645025, LM645030, KX036365, KX036367, LM645029, LM645022, LM645024, MN233912, MN233920, MN233909, MN233923, MN233911, MN233907, MN233908, MN233906, MN233955, MN234056, MK540996, MK540980, MK541011, MK541002, MK541004, MK541009, MK540998, MK540999, MK541000, MK541001, MK541003, MK541006, MK541010, MK541007, MK540997, MK541008, MK540974, MK540970, MK540960, MK540942, MK540937, MK540949, MK540952, MK540945, MK540941, MK540910, MK540908, MK540906, MK540909, MK540991, MK540994, MK540958, MK540962, MK540961, MK540973, LT909527, AF499686, LN713665, LN713666, LN713669, LN713670, LT993239, DD161903, DD161904, DD161905, DD161906, DI035301, DI043469, DI046747, DI084725, M31046, MK111075, LT993238, MK111077, MK111076, MK111078, LT993245, EF206709, EF206708, EF206720, EF206710, EF206711, EF206712, EF206713, EF206714, EF206715, EF206716, LN713542, LN713543, LN713545, LN713546, LN713567, LN713568, LN713569, LN713570, LN713571, LN713576, LN713577, LN713578, LN713579, LN713580, LN713581, LN713582, LN713583, LN713584, LN713585, LN713586, LN713587, LN713588, LN713589, LN713590, LN713591, LN713592, LN713593, LN713594, LN713595, LN713596, LN713597, LN713598, LN713599, LN713600, LN713601, LN713602, LN713603, LN713604, LN713605, LN713606, LN713607, LN713608, LN713609, LN713610, LN713611, LN713612, LN713613, LN713614, LN713615, LN713616, LN713617, LN713618, LN713619, LN713620, LN713621, LN713622, LN713623, LN713624, LN713625, LN713626, LN713627, LN713628, LN713629, LN713635, LN713636, LN713652, LN713653, LN713654, LN713655, LN713656, EF206719, LN713648, LN713649, LN713651, LT993236, LT993244, EU182346, FJ913470, LR812030, LR812031, LR812025, EF206707, LT993240, LN713552, LN713553, LN713554, LN713555, LN713556, LN713557, LN713558, LN713572, LN713573, LN713574, LN713575, LR812032, LR812029, LT993237, LN713647, LN713667, LN713668, LR812033, LN713634, LT993235, LN713633, MK111080, MK111079, LN713561, LN713562, LN713563, LN713564, LN713565, LN713566, LT993233, LN713650, LT993242, LN713631, LN713632, LT993241, LN713630, LN713662, LN713663, LN713664, LT993234, EU886633, EU886634, EU886631, EU886636, EU886632, LN713544, EU877071, EU877068, EU877069, EU877070, EU877067, LN713549, LN713551, LN713548, LN713547, LN713550, LT993246, EF206718, LN713659, LN713661, LN713658, LN713657, LN713660, EU886635, LN713559, LN713560, LN713643, LN713644, LN713645, LN713646, LN713639, LN713640, LN713641, LN713642, LN713637, LN713638, M13215, KX148110, AB839170, AB839169, FJ577895, GQ918139, LC325820, MG458315, LT909543, KX148215, KY860604, MK598340, MK598338, MK598342, MK598341, MK598344, KX148182, KY860598, KY860601, KX148165, KX148162, KX148163, KX148166, KX148169, KX148174, LT909533, KX148168, KY860608, KY860587, KY860585, MG458309, KX148223, MK760754, MK760696, MK760767, MK760736, MK760737, MK760730, MK760711, MK760717, MK760714, MK760718, MK760746, MK760727, KX148239, MK760713, MF172976, KX148175, LC571945, FJ959397, HQ317918, KR906746, MK760755, KX148269, KR906752, AB810256, KX148178, KX148256, KX148258, JN786878, KX148268, MF143331, MK540786, MK540777, MF143239, MF143243, MF143211, MF143195, MF143221, MF143199, MF143216, MF143235, MN418184, KY026415, MF143222, MF143253, MF143254, MF143249, MF143236, MF143240, MF143245, MF143246, MF143237, MF143238, MF143250, MF143252, MF143248, MF143251, MF143247, MF143241, MF143305, MK540677, MK540672, MG562583, MG562591, MG562592, MG562581, MG562578, MG562601, MG562572, MG562520, MG562600, MF143266, MF143349, MF143273, MF143275, MF143269, MF143270, MF143259, MF143261, MF143262, MF143265, MF143271, MF143272, MF143274, MF143264, MF143260, MF143355, MF143350, MF143367, MF143358, MF143344, MF143347, MF143351, MF143361, MF143365, MF143399, MF143356, MF143352, MF143357, MF143363, MF143371, MF143354, MF143391, MF143368, MF143345, MF143228, MF143224, MF143229, MF143226, MF143234, MF143231, MF143232, MF143233, MF143223, MF143225, MF143227, MG562557, MF143343, MF143342, MF143390, MK540715, MK540722, MN418164, MG562608, MG562586, MG562553, MF143395, MF143415, MG562609, MK540826, MK540804, MK540807, MK540856, MK540866, MK540870, MK540833, MK540805, MK540863, MK540825, MK540828, MK540829, MK540865, MK540808, MK540867, MK540823, MK540857, MK540871, MK540816, MK540876, MK540814, MK540810, MK540830, MK540818, MK540843, MK540803, MK540875, MK540861, MK540877, MK540864, MK540846, MK540801, MK540811, MK540812, MK540859, MK540815, MK540800, MK540836, MK540847, MK540848, MK540839, MK540841, MK540851, MK540862, MK540844, MK540819, MK540835, MK540822, MK540860, MK540840, MK540834, MK540817, MK540873, MK540837, MK540869, MK540820, MK540880, MK540878, MK540881, MK540874, MK540821, MK540813, MK540854, MK540832, MK540831, MF143255, MN418183, MN418169, MG562569, MK540747, MK540701, MG562564, MK540659, MK540742, MK540774, MG562577, MG562574, MG562556, MF143374, MG562566, MG562555, MG562561, KY026479, MF143287, MF143404, MF143398, MF143412, MF143386, MF143387, MF143396, MF143403, MF143394, MN418159, MN418157, MG562534, MG562528, MG562554, MK540780, MG562548, MK540743, MG562540, MG562582, MG562542, MG562541, MK540680, MG562598, MK540699, MK540723, MK540745, MK540690, MG562524, MK540671, MK540886, MK540662, MK540661, MK540668, MK540883, MK540666, MK540678, MK540885, MK540663, MK540667, MG562531, MF143285, MN418151, MN418152, MF143369, MF143339, MF143360, MF143364, MF143381, MF143378, MF143413, MF143409, MF143407, MF143389, MF143376, MG562536, MF143276, MF143263, MF143289, MF143268, MF143281, MF143267, MF143372, MF143410, MF143383, MF143408, MF143379, MF143380, MF143382, MF143388, MF143359, MF143370, MF143348, MF143353, MF143362, MF143256, KY026483, MK540849, MK540842, MK540809, MF143303, KY026424, KY026426, MK540686, MK540681, MK540683, MK540658, MK540673, MK540670, MK540676, MF143257, MF143298, KY026419, MK540669, MK540757, MF143325, MF143292, MK540754, MF143384, MF143402, MK540852, MK540660, MF143336, MG562568, MK540767, MG562585, MG562559, MF143373, MG562593, MG562567, MG562570, MK540771, MG562558, MF143288, MF143375, MF143406, MF143283, MF143377, MG562584, MG562604, MK540687, MK540746, MK540753, MF143314, MF143346, MF143244, MF143309, MF143310, JQ685956, JQ685940, JQ685969, JQ685962, JQ685959, JQ685893, JQ685904, JQ685958, JQ685923, JQ685966, JQ685935, JQ685930, JQ685927, JQ685949, JQ685897, JQ685912, JQ685939, JQ685892, JQ685908, JQ685928, JQ685976, JQ685972, JQ685937, JQ685896, JQ685948, JQ685977, JQ685914, JQ685924, MK598371, MK598394, MK598364, MK598355, MK598351, MK598379, MK598392, MK598397, MK598389, MK598396, MK598376, MK598372, MK598370, MK598393, MK598385, MK598386, MK598382, MK598384, MK598387, MK598352, MK598360, MK598369, MK598367, MK598377, MK598353, MK598356, MK598357, MK598358, MK598363, MK598373, MK598375, MK598354, MK598365, MK598366, MK598359, MK598391, MK598388, MK598368, MK598362, MK598383, MK598390, MK598380, KX148127, LT909538, KX148147, KX148133, KX148130, KX148119, KX148122, KX148124, LT909532, KX148126, MK598350, MK598347, MK598348, AB044824, KX148199, LT909542, LT909535, KR906753, MK760756, KY982922, KY210274, KY210269, KY210270, KR906776, KR906789, JX473839, MT454643, KY210309, KY210296, KR906781, KR906773, KY210230, KY210224, KR534250, KR534236, KR534237, KY210303, KY210248, KR534235, KY210297, KR906792, KY210305, KR906738, KR906742, KY210300, KR906787, KY210251, KR906755, KR906771, KY210231, KY210232, KY210235, KR534245, KR534246, KR534248, KY210239, KY210238, KR534247, KR534254, KY210291, KY210301, KY210307, KY210247, KY210272, KR534217, KR534218, KR534220, KY210265, KY210288, KY210286, KY210258, KR906780, KY210298, KY210250, KY210289, KY210302, KY210290, KR906760, KR906782, KR906778, KY210236, KY210234, KY210229, KY210292, KR534233, KY210278, KY210293, KR534244, KY210241, KY210243, KY210220, KY210249, KY210299, KY210226, KR906737, KY210244, KY210267, KR534232, KR534252, KY210252, KR906790, KY210311, KY210284, KY210242, KY210255, KY210256, KY210266, KY210304, KY210294, KR906784, KY210237, KY210233, KY210261, KY210277, KY210283, KR534234, KR534251, KY210281, KY210306, KR906734, KY210254, KR906785, KR906774, KY210227, KY210310, KR906743, KR906770, KY210280, KY210240, KY210246, KR906744, KR906765, KR906763, MT454653, MT454654, MT454641, MT454636, MT454639, MT454635, MT454652, KY912036, KC252634, MK567666, MN234032, KY026465, KY026464, KY026434, KY026470, KY026471, KY026432, KY026467, KY026466, KY026468, KY026461, KY026462, KY026455, KY026477, KY026433, KY026437, KY026439, KY026450, KY026452, KY026456, KY026431, KY026457, KY026459, KY026449, KY026438, KY026422, KY026425, KY026430, KY026436, KY026453, KY026423, KY026428, KY026454, KY026446, KY026458, KY026440, KY026442, KY026443, KY026444, KY026445, KY026463, KY026472, KY026473, KY026474, KY026475, KY026429, EU311738, KY026451, KY026435

**SINV VIPR Database All**

MF589985, MF409177, HV228506, HM147984, AF103734, MG679379, MF459683, JX570540, U38304, MF409178, MT270144, MF543016, MT270145, MH212167, M69205, MG779535, KT121617, KT121699, KT121605, BD269911, BD269910, KT121609, KT121606, KT121717, KT121710, KT121706, KT121696, KT121695, KT121688, KT121684, KT121681, KT121680, KT121675, KT121674, KT121673, KT121669, KT121668, KT121666, KT121662, KT121693, KT121671, KT121635, KT121634, KT121627, KT121616, KT121582, MG779533, GU361116, KT121651, KT121645, MT121982, MK440626, U38305, KF737350, MG495620, MG679380, MG679373, MG679376, JQ771799, JQ771798, JQ771797, JQ771795, MG679377, JQ771794, KY616988, JQ771793, KY616986, KY616984, KY616985, MG779534, MK045250, MK045246, MK045247, MN389434, MN389435, MK045240, MK045238, MK045224, MK045225, MK045231, MK045229, MK045230, MK045232, MK045236, MK045227, MK045228, MK045239, MK045242, MK045241, MK045245, MK045249, MK045252, MK045257, MK045256, MK045254, MK045243, MK045251, MK045255, MK045253, MK045244, MK045248

**BUNV VIPR database All**

KU159766, KT313717, KT313720, KT313723, KT313726, KT313735, KT313738, MH484279, KM496335, KT313759, KT313732, KT313741, KT313729, KT313744, KT313747, KT313750, KT313753, KT313756, KU661984, KT313698, KT313701, KT313713, KT313704, KT313710, KT313707, JX846603, KM507323, KC168048, MH299974, JX846597, JX846600, KJ187038, KU746871, KX100147, KX100153, KX100138, KX100135, MH166879, KX100144, KX100141, KX100123, KX100150, KX100126, KX100108, KC436106, MH484342, KX100120, MH484321, MF066368, MH484303, MH484324, MG828823, MH484333, FJ943510, FJ943509, MH484294, KX100132, KP063892, KP063898, KP063895, KX100114, KX100111, MH484312, MG544835, KX100117, KX100129, KX100105, KY910429, KJ710425, JX846606, MH484285, MH370824, MH484330, KM507336, KM507334, KM507335, JX857327, JX857330, MH484288, JX857324, NC_001925, X14383, KM507337, KM507338, KC608152, JX857318, JX857321, KJ716850, KC608149, KF234075, MH484282, MH370821
