# Supplementary material for: Extensive C->U transition biases in the genomes of a wide range of mammalian RNA viruses; potential associations with transcriptional mutations, damage- or host-mediated editing of viral RNA
Source: PLoS Pathog. 2021 Jun 1;17(6):e1009596. doi: 10.1371/journal.ppat.1009596 (PMC8195396; doi:10.1371/journal.ppat.1009596)
Supplement: S2 Table — (DOCX) [file ppat.1009596.s002.docx]

TABLE S2

COMPOSITIONAL FEATURES OF RNA VIRUS SEQUENCE DATASETS USED IN THE STUDY

|  |  |  | |  |  |  | **Base imbalance^3^** | | **Dinucleotide Rep.^4^** | |  |  | **Transition Asymm^5^** | | **Normalised Asymm^6^** | |
| --- | --- | --- | --- | --- | --- | --- | --- | --- | --- | --- | --- | --- | --- | --- | --- | --- |
| **Virus^1^** | **Family** | | **Polarity** | **n** | **MPD^2^** | **G+C** | **C/G Asymm** | **U/A** | **CpG** | **UpA** | **MFE** | **MFED** | **rG->A** | **rC->U** | **nG->A** | **nG->U** |
|  |  | |  |  |  |  |  |  |  |  |  |  |  |  |  |  |
| BUNV | *Orthobunyaviridae* | | *-* | 92 | 0.226 | 0.339 | -0.120 | -0.208 | 0.259 | 0.819 | -54.8 | 0.9% | 0.592 | 0.258 | 1.063 | 0.541 |
| HPeV-3 | *Picornaviridae* | | *+* | 181 | 0.092 | 0.390 | -0.107 | -0.111 | 0.174 | 0.757 | -51.9 | 1.6% | 0.698 | 0.596 | 1.107 | 0.938 |
| CHIKV | *Togaviridae* | | *+* | 245 | 0.042 | 0.508 | -0.023 | -0.301 | 0.834 | 0.873 | -85.6 | 5.3% | 0.919 | 1.213 | 1.028 | 0.957 |
| SINV | *Togaviridae* | | *+* | 101 | 0.037 | 0.509 | 0.008 | -0.285 | 0.916 | 0.785 | -80.6 | 1.9% | 0.898 | 1.403 | 1.010 | 1.097 |
| EBOV | *Filoviridae* | | *-* | 200 | 0.001 | 0.413 | 0.086 | -0.154 | 0.591 | 0.730 | -68.1 | 2.2% | 0.840 | 0.890 | 1.353 | 1.118 |
| MeV | *Paramyxoviridae* | | *-* | 224 | 0.039 | 0.476 | 0.026 | -0.199 | 0.488 | 0.741 | -76.9 | -0.1% | 0.945 | 1.161 | 1.189 | 1.140 |
| HEV | *Hepeviridae* | | *+* | 100 | 0.187 | 0.556 | 0.138 | 0.408 | 0.796 | 0.950 | -98.5 | 3.9% | 2.709 | 1.430 | 1.910 | 1.231 |
| BVDV | *Flaviviridae* | | *+* | 123 | 0.178 | 0.456 | -0.226 | -0.311 | 0.377 | 0.901 | -48.1 | 0.7% | 0.788 | 0.929 | 0.950 | 1.305 |
| IAV_seg1-3 | *Orthomyxoviridae* | | *-* | 1340 | 0.138 | 0.431 | -0.204 | -0.338 | 0.428 | 0.578 | -63.6 | 0.6% | 0.614 | 0.974 | 0.850 | 1.309 |
| Porcine_KoV | *Picornaviridae* | | *+* | 138 | 0.121 | 0.520 | 0.537 | 0.341 | 0.640 | 0.570 | -84.1 | 16.9% | 0.925 | 1.796 | 0.865 | 1.387 |
| Rabies | *Rhabdoviridae* | | *-* | 2128 | 0.129 | 0.449 | -0.025 | -0.019 | 0.450 | 0.700 | -78.0 | -0.1% | 1.049 | 1.079 | 1.275 | 1.479 |
| EV-A71 | *Picornaviridae* | | *+* | 1161 | 0.148 | 0.464 | -0.208 | -0.325 | 0.524 | 0.777 | -63.7 | 0.7% | 0.725 | 1.295 | 0.822 | 1.509 |
| DENV1 | *Flaviviridae* | | *+* | 1557 | 0.066 | 0.464 | -0.208 | -0.325 | 0.424 | 0.583 | -59.4 | 1.8% | 0.745 | 1.542 | 0.935 | 1.575 |
| HPgV-1 | *Flaviviridae* | | *+* | 100 | 0.122 | 0.592 | -0.150 | 0.292 | 0.697 | 0.669 | -98.9 | 12.0% | 2.477 | 2.055 | 1.181 | 1.765 |
| RSV-A | *Pneumoviridae* | | *-* | 100 | 0.026 | 0.333 | 0.135 | -0.285 | 0.230 | 0.892 | -74.7 | 1.9% | 0.524 | 1.067 | 1.349 | 1.771 |
| OC43 | *Coronaviridae* | | *+* | 113 | 0.010 | 0.370 | -0.300 | 0.316 | 0.474 | 0.936 | -62.7 | 17.7% | 0.812 | 0.757 | 1.023 | 1.851 |
| JEV | *Flaviviridae* | | *+* | 62 | 0.088 | 0.516 | -0.193 | -0.236 | 0.603 | 0.531 | -69.5 | 1.4% | 0.971 | 2.156 | 0.924 | 1.886 |
| MNV | *Caliciviridae* | | *+* | 63 | 0.102 | 0.569 | -0.017 | -0.009 | 0.621 | 0.487 | -85.1 | 7.1% | 1.539 | 2.587 | 1.191 | 2.079 |
| HCV-3a | *Flaviviridae* | | *+* | 820 | 0.085 | 0.557 | 0.033 | 0.090 | 0.715 | 0.825 | -83.3 | 8.7% | 1.372 | 2.978 | 0.998 | 2.110 |
| Canine_KoV | *Picornaviridae* | | *+* | 25 | 0.125 | 0.584 | 0.808 | 0.122 | 0.738 | 0.428 | -88.4 | 17.8% | 0.883 | 3.690 | 1.910 | 2.125 |
| HCV-2a | *Flaviviridae* | | *+* | 51 | 0.109 | 0.578 | 0.060 | 0.045 | 0.694 | 0.758 | -95.0 | 7.7% | 1.763 | 2.851 | 1.224 | 2.149 |
| HKU1 | *Coronaviridae* | | *+* | 27 | 0.002 | 0.320 | -0.316 | 0.446 | 0.453 | 0.958 | -50.2 | 9.6% | 1.250 | 0.692 | 1.841 | 2.163 |
| TGEV | *Coronaviridae* | | *+* | 38 | 0.022 | 0.375 | -0.188 | 0.126 | 0.474 | 0.818 | -57.0 | 8.8% | 0.548 | 1.091 | 0.782 | 2.265 |
| FMDV-O | *Picornaviridae* | | *+* | 246 | 0.106 | 0.536 | 0.094 | -0.166 | 0.815 | 0.437 | -77.6 | 11.7% | 1.059 | 3.095 | 1.029 | 2.266 |
| HNoV_GGII | *Caliciviridae* | | *+* | 100 | 0.189 | 0.496 | -0.024 | -0.268 | 0.439 | 0.563 | -83.2 | 1.6% | 0.844 | 1.990 | 0.746 | 2.492 |
| OC43 | *Coronaviridae* | | *+* | 178 | 0.008 | 0.366 | -0.290 | 0.305 | 0.466 | 0.925 | -62.1 | 17.5% | 1.543 | 1.034 | 1.992 | 2.503 |
| FMDV-A | *Picornaviridae* | | *+* | 98 | 0.112 | 0.536 | 0.094 | -0.151 | 0.819 | 0.478 | -88.2 | 12.1% | 1.015 | 3.118 | 0.997 | 2.559 |
| NL63 | *Coronaviridae* | | *+* | 61 | 0.009 | 0.345 | -0.275 | 0.490 | 0.413 | 0.876 | -50.2 | 8.6% | 1.144 | 0.945 | 1.510 | 2.654 |
| HCV-1b | *Flaviviridae* | | *+* | 102 | 0.094 | 0.587 | 0.060 | 0.044 | 0.736 | 0.765 | -88.3 | 8.5% | 1.810 | 3.991 | 1.238 | 2.905 |
| 229E_Camel | *Coronaviridae* | | *+* | 33 | 0.002 | 0.384 | -0.226 | 0.281 | 0.500 | 0.810 | -59.6 | 10.4% | 0.577 | 1.398 | 0.720 | 2.906 |
| 229E_Human | *Coronaviridae* | | *+* | 26 | 0.007 | 0.381 | -0.228 | 0.284 | 0.490 | 0.794 | -53.7 | 10.4% | 0.667 | 1.389 | 0.842 | 2.981 |
| MERS-CoV | *Coronaviridae* | | *+* | 26 | 0.005 | 0.412 | -0.033 | 0.244 | 0.559 | 0.897 | -59.6 | 15.7% | 1.104 | 1.840 | 1.386 | 2.986 |
| HCV-1a | *Flaviviridae* | | *+* | 355 | 0.083 | 0.587 | 0.070 | 0.065 | 0.720 | 0.777 | -88.3 | 9.0% | 2.094 | 4.734 | 1.441 | 3.019 |
| RUBV | *Matonaviridae* | | *+* | 73 | 5.42% | 0.696 | 0.260 | 0.040 | 1.057 | 0.732 | -131.0 | 3.2% | 1.258 | 3.596 | 1.258 | 3.596 |
| SARS-CoV | *Coronaviridae* | | *+* | 22 | 0.000 | 0.408 | -0.038 | 0.082 | 0.465 | 0.800 | -61.5 | 13.5% | 0.667 | 2.714 | 0.911 | 4.177 |
| SARS-CoV-2 | *Coronaviridae* | | *+* | 17550 | 0.000 | 0.379 | -0.066 | 0.077 | 0.400 | 0.827 | -58.7 | 15.1% | 1.562 | 7.481 | 1.564 | 7.486 |

^1^Abbreviations: See Table 1

^2^MPD: mean pairwise uncorrected nucleotide distance

^3^Excess of the frequency of C over the frequency of G, or U over A

^4^Representation expressed as the observed frequency divided by expected frequency based on mononucleotide composition

^5^Ratio of G->A transitions to A->G transitions or C->U transitions to U->C transitions

^6^Corrected ratio based on nucleotide composition (see Results text).
