# Supplementary material for: Extensive C->U transition biases in the genomes of a wide range of mammalian RNA viruses; potential associations with transcriptional mutations, damage- or host-mediated editing of viral RNA
Source: PLoS Pathog. 2021 Jun 1;17(6):e1009596. doi: 10.1371/journal.ppat.1009596 (PMC8195396; doi:10.1371/journal.ppat.1009596)
Supplement: S1 Fig — (DOCX) [file ppat.1009596.s004.docx]

FIGURE S1

MUTATION FREQUENCIES OF RNA VIRUSES ANALYSED IN THE STUDY


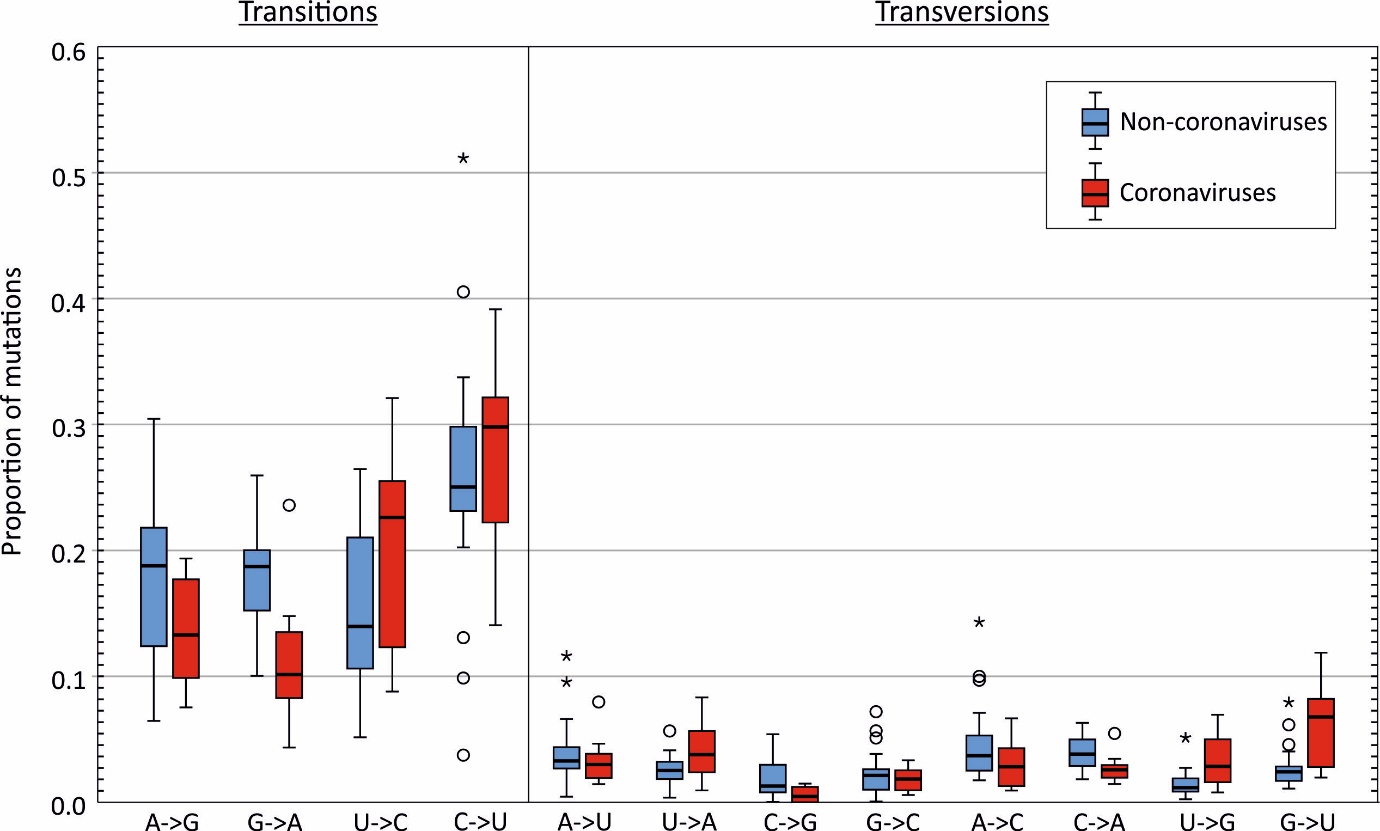


Relative frequencies of each mutation type expressed as a percentage of all changes (y-axis) in the 36 RNA virus alignments at sites showing <5% heterogeneity. Tukey box plots show maximum, upper interquartile range (IQR), median, lower IQR and minimum values of each distribution.
