# Supplementary material for: Extensive C->U transition biases in the genomes of a wide range of mammalian RNA viruses; potential associations with transcriptional mutations, damage- or host-mediated editing of viral RNA
Source: PLoS Pathog. 2021 Jun 1;17(6):e1009596. doi: 10.1371/journal.ppat.1009596 (PMC8195396; doi:10.1371/journal.ppat.1009596)
Supplement: S2 Fig — (DOCX) [file ppat.1009596.s005.docx]

FIGURE S2

UNROOTED PHYLOGENIES OF SEQUENCE ALIGNMENTS USED FOR HOMOPLASY ANALYSIS


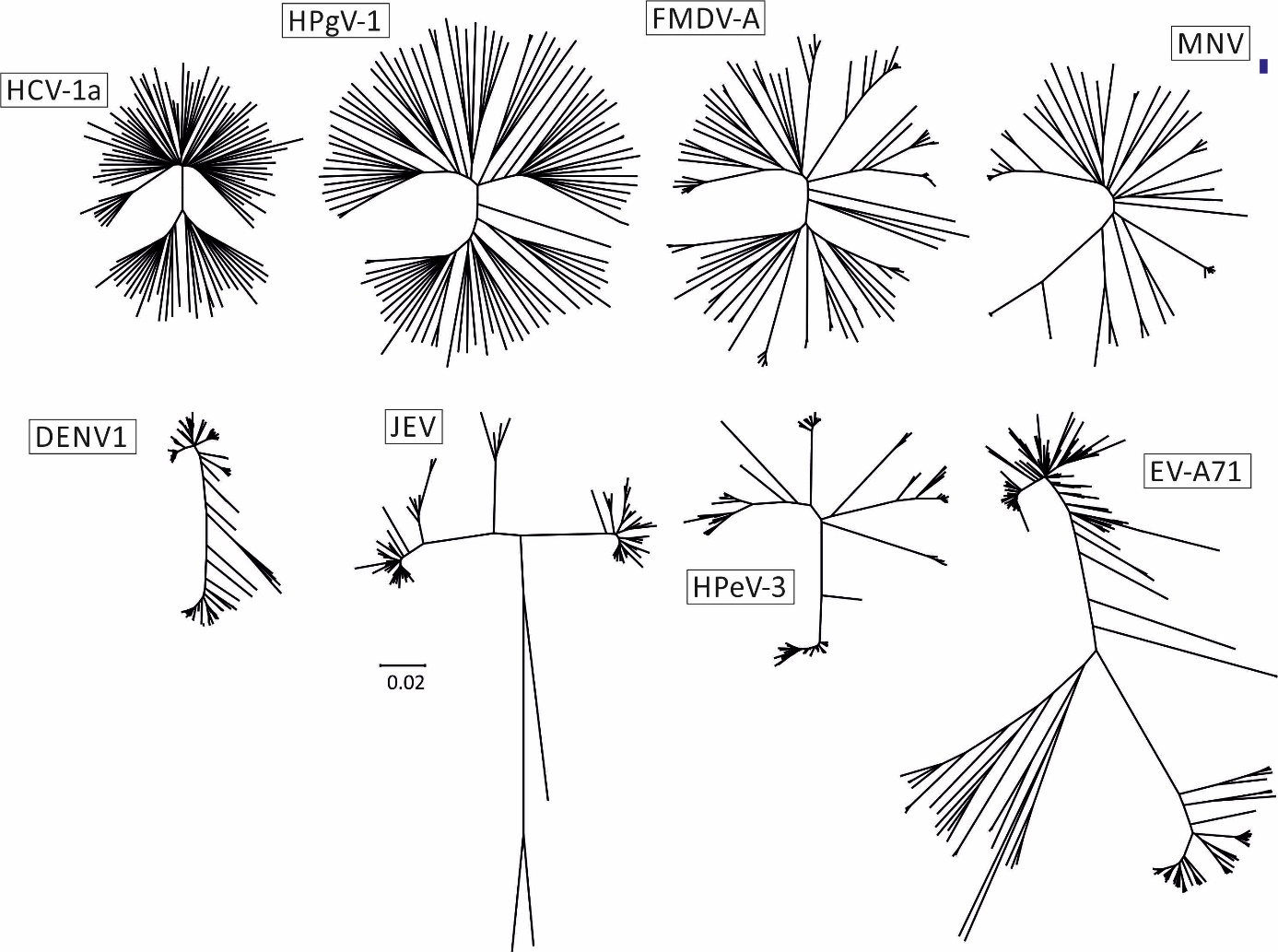


Neighbour-joining trees of coding region sequences used for lineage through time analysis plotted to the same scale (see scale). For comparability of the depicted trees, randomly selected subsets of 100 sequences were used for the larger HCV-1a, DENV1 and EV-A71 sequence alignments.
