# Supplementary material for: Extensive C->U transition biases in the genomes of a wide range of mammalian RNA viruses; potential associations with transcriptional mutations, damage- or host-mediated editing of viral RNA
Source: PLoS Pathog. 2021 Jun 1;17(6):e1009596. doi: 10.1371/journal.ppat.1009596 (PMC8195396; doi:10.1371/journal.ppat.1009596)
Supplement: S3 Fig — (DOCX) [file ppat.1009596.s006.docx]

FIGURE S3

LIENEAGE THROUGH TIME PLOT FOR SEQUENCE ALIGNMENTS USED FOR HOMOPLASY ANALYSIS


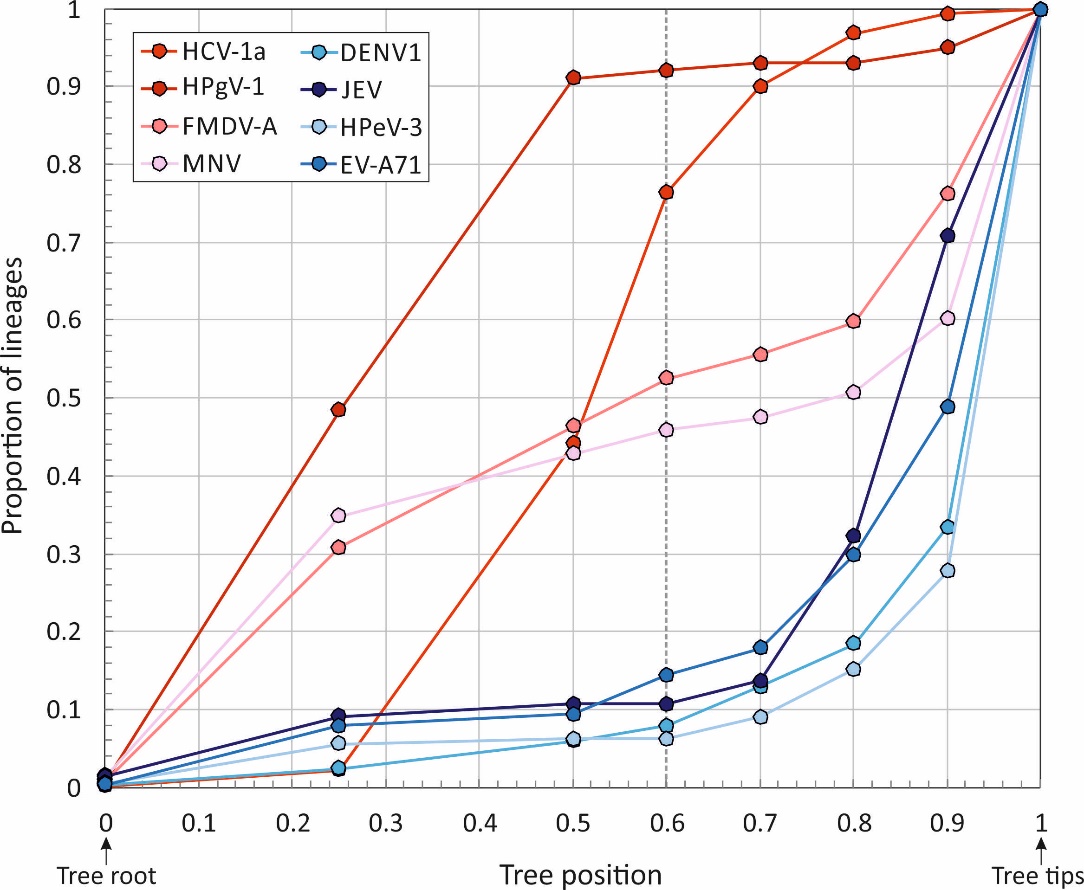


Proportion of total lineages (y-axis) at different positions in neighbour joining phylogenetic trees of virus alignments used for homoplasy analysis (x-axis). Virus groups were divided into those with high (red) and unbiased (blue) C->U / U->C transition asymmetries.
